# Succeed to culture a novel lineage symbiotic bacterium of *Mollicutes* which widely found in arthropods intestine uncovers the potential double-edged sword ecological function

**DOI:** 10.3389/fmicb.2024.1458382

**Published:** 2024-10-18

**Authors:** Lingyu Zhang, Qi Chen, Shenzheng Zeng, Zhixuan Deng, Zhongcheng Liu, Xuanting Li, Qilu Hou, Renjun Zhou, Shicheng Bao, Dongwei Hou, Shaoping Weng, Jianguo He, Zhijian Huang

**Affiliations:** ^1^State Key Laboratory of Biocontrol, School of Life Sciences, School of Marine Sciences, Sun Yat-sen University, Guangzhou, China; ^2^Southern Marine Sciences and Engineering Guangdong Laboratory (Zhuhai), School of Marine Sciences, Sun Yat-sen University, Zhuhai, China

**Keywords:** symbiotic bacteria, intestinal microbiota, *Candidatus* bacilloplasma, *Enteroplasmatales* ord. nov., *Penaeus vannamei*

## Abstract

Symbiotic gut bacteria play crucial role in host health. Symbionts are widely distributed in arthropod intestines, but their ecological functions are poorly understood due to the inability to cultivate them. Members of *Candidatus* Bacilliplasma (CB) are widely distributed in crustacean intestine and maybe commensals with hosts, but the paucity of pure cultures has limited further insights into their physiologies and functions. Here, four strains of representative CB bacteria in shrimp intestine were successfully isolated and identified as members of a novel Order in the Phylum *Mycoplasmatota*. Through genome assembly, the circular genome maps of the four strains were obtained, and the number of coding genes ranged from 1,886 to 1,980. Genomic analysis suggested that the bacteria were missing genes for many critical pathways including the TCA cycle and biosynthesis pathways for amino acids and coenzyme factors. The analysis of 16S amplification data showed that *Shewanella, Pseudomonas* and CB were the dominant at the genera level in the intestine of *Penaeus vannamei*. Ecological functional experiments revealed that the strains were symbionts and colonized shrimp intestines. Our valued findings can greatly enhance our understanding and provides new insights into the potentially significant role of uncultured symbiotic bacteria in modulating host health.

## 1 Introduction

Symbiotic gut microorganisms are critical factors that influence physiological, immunological, and metabolic functions of hosts (Gao et al., 2021; Koch and Schmid-Hempel, 2011; Emery et al., 2017). Increasing evidences have recently suggested that symbiotic gut bacteria are keystone organisms that play fundamental roles in gut ecosystem and host health (Akami et al., 2019; Schroeder and Bäckhed, 2016). *Akkermansia muciniphila* is one such important gut symbiont in human that helps maintain metabolic homeostasis and is currently recommended as a probiotic to aid clinical obesity, diabetes, and liver diseases (Cani and de Vos, 2017; Plovier et al., 2017; Derrien et al., 2004). By contrast, *Enterobacter cloacae* is a gut bacterium that is causative for the development of obesity and insulin resistance in animals (Fei and Zhao, 2013). Furthermore, the gut symbiont *Bacillus siamensis* LF4 effectively induces intestinal antimicrobial peptide expression in spotted seabass (Liu et al., 2023). In addition to the above, *Candidatus* Hepatoplasma crinochetorum is a hepatopancreatic symbiont of *Porcellio scaber* that might have enabled isopods to colonize new habitats and extend their geographic distributions (Wang et al., 2004). Likewise, genomic analysis of *Candidatus* Termititenax bacterial symbionts of termite guts revealed that the bacteria may mutualistically interact with hosts via cellulose digestion (Utami et al., 2019), and other reports showed that suggest that symbiotic bacteria contribute to the digestion of *Coptotermes formosanus* (Dar et al., 2022; Dar et al., 2024). Owing to their greatly reduced, gene-poor genomes, these commonly symbiotic bacteria are not cultivable outside of their host and pure cultures of many of these symbiotic microorganisms cannot be obtained, their functions can only be predicted rather than confirmed, limiting research into symbiotic bacterial functions.

Microorganisms are the most abundant organisms on Earth and are ubiquitous in a variety of environments but over 99% of bacterial and archaeal species have not been isolated, with the uncultured organisms referred to as “microbial dark matter” (Yarza et al., 2014). Uncultured microorganisms often comprise phylogenetically divergent and deep-branching groups and dominate non-human environments, and their physiologies may impact Earth ecosystems (Lloyd et al., 2018). Indeed, numerous studies have revealed important potential functions of uncultured taxa in specific environments. For example, Marine Group II *Euryarchaea* are abundant in some marine environments and are considered important for degrading high molecular weight organic matter (Martin-Cuadrado et al., 2015). Furthermore, analysis of metagenome-assembled genomes (MAGs) indicated that members of three uncultured novel actinomycetes classes rely on hydrogen to reduce carbon dioxide to acetic acid, representing a previously unknown characteristic of the group (Jiao et al., 2021). Likewise, Hua et al. reconstructed 14 microbial genomes with methane or alkane metabolic capacity from Yunnan and Tibet hot springs, revealing their important ecological functions in geochemical cycles (Hua et al., 2019). Thus, uncultured microorganisms comprise a high abundance of natural communities, and their encoded genes, novel metabolic pathways, and physiological characteristics potentially represent significant microbial resources with great application prospects.

*Candidatus* Bacilloplasma (referred to as CB bacteria) is a previously reported novel bacterial genus belonging to the class *Mollicutes* of the phylum *Mycoplasmatota*, which is symbiotic bacteria in the hindguts of the terrestrial isopod crustacean *Porcellio scaber* (Kostanjsek et al., 2007). Attempts to cultivate these bacteria and determine their phenotypical properties have been unsuccessful, and thus *Candidatus* status has been given for the lineage that was documented in the LPSN (List of Prokaryotic names with Standing in Nomenclature).^1^ To maintain consistency with naming conventions, the proposed *Candidatus* Bacilloplasma epiphet was amended to *Candidatus* Bacilliplasma by the ICSP (International Committee on Systematics of Prokaryotes) in 2020 (Oren et al., 2020). Fifteen CB representative phylotypes have been identified by searches of the comprehensive microbial taxonomic databases EzBioCloud and Silva. Furthermore, CB bacteria have been detected in the intestines of *Gillichthys mirabilis* (Bano et al., 2007) and the stomach of the yellow catfish (*Pelteobagrus fulvidraco*) (Wu et al., 2012). In addition, CB were among the abundant genera in the intestines of shrimp with white feces syndrome (WFS) (Hou et al., 2018). Moreover, CB bacteria have been identified as “indigenous” flora in *Eriocheir sinensis* intestines (Wang C. H. et al., 2019). CB bacteria have also been identified as the most abundant genus in the intestine of crayfish within integrated rice-crayfish systems and were a core population in the intestines (Wang et al., 2021). Thus, CB are widely distributed and highly abundant in the intestines of arthropods and fishes.

*Penaeus vannamei* is one of the most important cultured crustacean species globally, with global production encompassing 4.4 million tons valued at over $26.7 billion and accounting for 80% of total cultured shrimp production (FAO, 2020). However, shrimp production is threatened by several diseases including acute hepatopancreatic necrosis disease (AHPND) (Lee et al., 2015), hepatopancreas necrosis syndrome (HPNS) (Huang et al., 2016), and WFS (Huang et al., 2020). Intestinal microbiota (IM) plays fundamental roles in regulating host metabolic homeostasis, physiology, and health (Jie et al., 2017; Just et al., 2018; Xiong et al., 2019). Indeed, some diseases are associated with disturbances of IM (Kassinen et al., 2007; Sivan et al., 2015). Significant increases in abundances of CB may contribute to WFS in shrimp (Hou et al., 2018). An additional study suggested that the relative abundances of *Vibrio*, CB, *Photobacterium*, and *Aeromonas* were overrepresented in WFS shrimp, suggesting a causality between WFS disease and intestinal flora dysbiosis of *P. vanmamei* (Huang et al., 2020). Furthermore, significantly decreases abundances of CB have been observed in the intestines of *P. vanmamei* with white spot syndrome virus (WSSV) (Wang J. et al., 2019). These studies suggest that changes in the abundances of important IM like CB in *P. vanmamei* are closely related to the occurrence of shrimp diseases. However, the successful cultivation of CB bacteria has yet to be achieved.

In the present study, the first representative strain of CB was isolated and subjected to polyphasic taxonomic identification. Four CB strains were identified as members of a novel order in the phylum *Mycoplasmatota*, followed by an analysis of their genomic characteristics and metabolic potential. A comprehensive exploration of the unique ecological function of CB bacteria in shrimp intestines was subsequently conducted. These results overall document the cultivation of a previously uncultured group of bacteria that are members of a novel Order in Phylum *Mycoplasmatota*. Further analysis of their microecology as symbionts provides new insights into their potentially significant roles in modulating host health.

## 2 Materials and methods

### 2.1 Bacterial isolation and cultivation

Intestinal samples were collected from shrimp in culture ponds in Jiangmen, Yangjiang, Zhuhai, Guangzhou City, China. Shrimp surfaces were sterilized with 70% ethanol, and intact intestines were aseptically dissected and placed into sterile 2 mL centrifuge tubes (Zeng et al., 2017). The 16S rRNA gene sequences of CB were used to predict the appropriate culture medium with the Know Media Database (KOMODO) platform (Oberhardt et al., 2015). Eight media were selected for optimizing bacterial culture including nutrient broth agar medium (NA), Columbia blood agar (CBA), tryptic soy agar (TSA), brain-heart infusion agar (BHIA), 1/10 strength nutrient broth agar medium (1/10 NA, 10-fold dilution of the original medium), anaerobic broth agar medium (AB), 1/10 strength Zobell Marine Broth 2216 agar medium (1/10 2216E), and 1/10 strength anaerobic broth agar medium (1/10 AB). The media were supplemented with resazurin solution (5 mg/L) and L-cysteine (50 mg/L). Shrimp intestines (0.5 g) were thoroughly ground with sterile saline (5 ml) in a sterile homogenizer to generate a suspension. Suspensions were serially diluted 100- and 1,000-fold, with 100 μL of each diluted suspension used to prepare plates for coating. Inoculated plates were incubated at 32°C for three days in an anaerobic incubator (COY-7150220, Michigan, USA). Individual colonies with distinct morphologies were then picked using sterilized wooden sticks and then repeatedly cultured and purified on solid NA medium until they were axenic. Strains were preserved at −80°C in 25% (v/v) glycerol. A total of 11 strains with 16S rRNA gene sequences similar to CB were isolated using the above methodology. Among these, four strains that were most similar to CB and exhibited rapid growth were selected for subsequent studies and identified as LVI A0006, LVI A0039, LVI A0075, and LVI A0078. Pleuropneumonia-like organisms broth medium (PPLO) (Hopebio, Shandong, China) was used for subsequent cultivation because strains grew slowly on NA medium.

### 2.2 Electron microscopy observations

Scanning electron microscope (SEM) observations were conducted by washing samples three times with sterile phosphate buffer saline (PBS) and fixing them in 2.5% glutaraldehyde in 0.1 M PBS at 4°C overnight. Samples were subsequently dehydrated in gradient ethanol solutions and dried in a critical point dryer (Samdri-PVT-3D, Porlant, USA) as previously described (Chen et al., 2015), but with slight modifications. The dried samples were viewed on a specimen stage with double-sided adhesive tape, coated with gold using a sputter coater (HITACHI E-1010, Tokyo, Japan), and observed using a SEM (ZEISS Crossbeam-550, Oberkohen, Germany).

Ultrathin-section electron microscopic observations were performed as previously described (Sekiguchi et al., 2003), but with slight modifications. Ultrathin sections (60–80 nm) of cells were cut with a frozen ultramicrotome (Leica UC6/FC6, Wetzlar, Germany) and mounted on copper grids. The samples were then examined using a transmission electron microscope (TEM) (JEOL, JEM-1400, Tokyo, Japan).

### 2.3 Physiological and biochemical characterization

The growth experiments of the four strains at different temperatures (4°C, 15°C, 25°C, 28°C, 32°C, 34°C, 37°C, 45°C), pH (4.0–10.0, 1.0 pH units ntervals), and NaCl (0%-8.0%, 1.0% intervals) concentration ranges were carried out according to the previous described methods (Ming et al., 2020). Catalase activity was detected based on bubble formation after the addition of a drop of 3% (v/v) H_2_O_2_ solution in the cultures. Oxidase activity was detected using an oxidase reagent (BioMérieux, Lyons, France) according to the manufacturer’s instructions. Other physiological and biochemical properties were evaluated using commercial API 20A kits (BioMérieux, Lyons, France). Gram-staining was conducted with a Gram-stain kit (Jiancheng, Nanjing, China). Cellular fatty acids were extracted, methylated, and analyzed using the Microbial Identification System (MIDI) and by following the manufacturer’s instructions (Sherlock Version 6.1; MIDI database: TSBA6) (Sasser, 1990).

### 2.4 Genome sequencing and characteristics

Genomic DNA was extracted using the sucrose, Tris, EDTA (SRE) method (Shepherd and McLay, 2011). DNA extracts were evaluated with agarose gel electrophoresis and quantified with a Qubit^®^ 2.0 Fluorometer (Thermo Scientific, Shanghai). The genomes of the four strains were sequenced using the PacBio Sequel and Illumina NovaSeq PE150 platforms at Novogene Bioinformatics Technology Co., Ltd (Beijing, China). To ensure the accuracy of subsequent analyses, low-quality reads (< 500 bp length) were removed to obtain clean data. Filtered reads were assembled using the Single Molecule Real-Time (SMRT) Link v5.0.1 software program (Ardui et al., 2018) and corrected with the Illumina sequencing data. Chromosome and plasmid sequences were identified and assembled into circular genomes. Gene functions were predicted by Kyoto Encyclopedia of Genes and Genomes (KEGG) (Kanehisa et al., 2006), Cluster of Orthologous Groups of proteins (COG) (Galperin et al., 2015), Pathogen Host Interactions Database (PHI-base) (Urban et al., 2015), Virulence Factors of Pathogenic Bacteria (VFDB) (Liu et al., 2022), Carbohydrate-Active enZYmes (CAZy) databases (Cantarel et al., 2009), Comprehensive Antibiotic Research Database (CARD) (Jia et al., 2017).

The genome relatedness values were calculated using multiple approaches including average nucleotide identity (ANI) (ANIm and ANIb) and tetranucleotide signatures (Tetra), calculated with the JSpeciesWS^2^ program (Richter et al., 2016). The average amino acid identity (AAI) values were calculated with the majorbio platform^3^ (Ren et al., 2022). In addition, DNA–DNA hybridization (DDH) values were calculated using the Genome-to-Genome Distance Calculator (GGDC)^4^ (Meier-Kolthoff et al., 2013).

### 2.5 Phylogenetic analyses

16S rRNA gene sequences were obtained from isolates and assembled using the DNAMAN 8.0 software program, followed by comparison against corresponding sequences of other cultured species in the EzBioCloud server^5^ (Yoon et al., 2017). To determine the phylogenetic relationships of strains LVI A0039, LVI A0006, LVI A0075, LVI A0078, and members of *Mollicutes*, multiple alignments of their 16S rRNA gene sequences were performed using the CLUSTAL_X software package (Thompson et al., 1997). Phylogenetic and molecular evolutionary analyses were then performed using the Molecular Evolutionary Genetics Analysis (MEGA) version 7.0 software package (Kumar et al., 2016). Phylogenetic dendrograms were generated with neighbor-joining, Maximum-Parsimony, and Maximum-Likelihood algorithms (Saitou and Nei, 1987; Fitch, 1971; Felsenstein, 1981). The Kimura two-parameter model (Kimura, 1980) was used to calculate evolutionary distances with the neighbor-joining and Maximum-Likelihood phylogenetic dendrograms. Bootstrap analysis was used to evaluate the topology of each tree with 1,000 replicates (Felsenstein, 1985).

To generate a genome-based phylogeny, genome sequences were retrieved from the NCBI database. Genomes were quality-checked using CheckM (Parks et al., 2015), and those with less than 80% completeness and more than 5% contamination were discarded. A total of 11 universal protein marker genes (*rpl*N, *rpl*O, *rpl*R, *rpl*V, *rpl*B, *rpl*C, *rpl*F, *rps*J, *rps*Q, *rps*C, and *rps*H) were extracted from the four genomes obtained in this study and the 27 genomes available for *Mollicutes*, 10 *Bacillota* phylum genomes, and seven from the phylum *Actinomycetota* using the AMPHORA2 software program (Wu and Scott, 2012). The genes were aligned using MUSCLE (Edgar, 2004) with 100 iterations to generate a phylogenomic tree. Poorly aligned regions were removed from the datasets using the Gblocks program (Castresana, 2000). Cleaned alignments were then concatenated using a perl script,^6^ followed by the generation of a Maximum-Likelihood phylogenetic tree using FastTree (Price et al., 2010) with default parameters.

### 2.6 Verification of strain ecological functions

To explore the functional role of Strain LVI A0039 in shrimp intestines, the strain was inoculated into *P. vannamei* intestines using reverse gavage and mixing with feed, followed by a series of experiments to verify the ecological function of the strain. Growth experiments and morphological observations were used to confirm that Strain LVI A0039 exhibited good growth and similar morphological characteristics as CB that were identified previously (Kostanjsek et al., 2007), followed by functional verification. To generate inoculum, Strain LVI A0039 was streaked on PPLO agar and incubated for 24 h. Colonies were picked from plates and inoculated into 100 mL PPLO liquid medium followed by incubation for 24 h. Absorbance was then measured as optical density (OD) at 600 nm, indicating an approximately 2 × 10^5^ CFUs⋅mL^–1^ cell density.

Shrimp with an average weight of 13.5 ± 0.5 g were collected from *P. vannamei* culture ponds in Guangzhou, Guangdong, China (23.02°N, 113.45°E). Shrimp were temporarily fed in the tank with 1% of their body weight twice a day. Shrimp were randomly divided into eight groups identified as I, II, III, IV, V, VI, VII, and VIII, with 100 shrimp in each group. The shrimp in the four groups (Groups I, II, III, and IV) were supplemented with strain LVI A0039 by reverse gavage twice, while the other four groups (Groups V, VI, VII, and VIII) were fed with strain inoculum mixed into feed for two days. Shrimp in Groups I and V received strain LVI A0039 without antibiotics, while shrimp in Groups II and VI received PBS without antibiotics. Shrimp in Groups III and VII received strain LVI A0039 and an antibiotic, while shrimp in Groups IV and VIII received PBS without antibiotics (Supplementary Figure 1 and Supplementary Table 1). Antibiotics were administered two days before shrimp were supplemented with strain inoculum for 400 mg⋅kg^–1^ commercial pelleted feed. Strain LVI A0039 and PBS supplementation was then conducted by reverse gavage and mixing into feed (Aranguren et al., 2010). After 12 h, the shrimp were supplemented with strain inoculum and fed commercial pelleted feed twice a day.

Samples were collected at 12 h, 24 h, 48 h, and 96 h. Shrimp surfaces were sterilized with 70% ethanol, and their intestines were aseptically dissected (Sriurairatana et al., 2014). Samples were immediately stored at −80°C before DNA extraction. To conduct fluorescent in situ hybridization (FISH) evaluation, nearly 1 cm of shrimp intestine was removed and soaked with 4% paraformaldehyde fixative, while nearly 1 cm of shrimp intestine was removed and soaked with 2.5% glutaraldehyde fixative for SEM observations (Chen et al., 2015).

### 2.7 Analysis of fluorescence in situ hybridization (FISH)

A specific oligonucleotide probe was designed to target strain LVI A0039 (FISHA0039) (5′-CTAATCACAATCTAGTGC-3′) and labeled it with Cyanine 3 (Cy3) at the 5′ end. The 16S rRNA gene was targeted with the specific oligonucleotide probe EUB 338 (5′-GCTGCCTCCCGTAGGAGT-3′) that was labeled with 5-carboxyfluorescein (5-FAM) at the 5′ end. Probes were synthesized at Wuhan Servicebio Technology Co., Ltd.

Shrimp intestines were removed and washed three times with PBS, followed by immediately placing them in fixed fluid (treated with diethyl pyrocarbonate) for 12 h. The samples were then dehydrated in an alcohol gradient, fixed with paraffin, and embedded. The paraffins were sliced with a slicer (RM2016, Shanghai, China) and then incubated for two hours in an oven (GSP-70, Tianjin) at 62°C. Soaked sections were dewaxed with xylene and ethanol, then hybridized as previously described (Foster et al., 2010), with slight modifications. The samples were analyzed with a confocal microscope (Leica TCS SP8 STED 3X, Wetzlar, Germany) using filter sets for DAPI, Cy3, and FAM.

### 2.8 16S rRNA gene amplicon sequencing and analysis

Total bacterial DNA from shrimp intestines was extracted using a DNeasy^®^ PowerSoil^®^ Pro Kit (Qiagen, Dusseldorf, Germany) following the manufacturer’s instructions with slight modifications. The PCR primers 338F and 806R (5′-ACTCCTACGGGAGGCAGCAG-3′ and 5′-GGACTACHVGGGTWTCTAAT-3′, respectively) were used for amplification since they have been broadly used for amplifying the V3-V4 hypervariable regions of bacterial 16S rRNA genes (Huang et al., 2021). PCR products were then purified using a Qiagen Gel Extraction Kit (Qiagen, Dusseldorf, Germany), and sequencing libraries were generated with indices using the TruSeq^®^ DNA PCR-Free Sample Preparation Kit (Illumina, California, USA) following the manufacturer’s recommendations. Finally, the amplicon concentrations were assessed with a Qubit@2.0 Fluorometer (Thermo Scientific, Shanghai), and their size distribution was evaluated with an Agilent 2100 Bioanalyzer (Agilent Technologies, Santa Clara, CA, USA). The library was then sequenced using the Illumina MiSeq platform (Illumina, San Diego, CA, USA) to generate 250 bp paired-ends at Novogene Co., Ltd (Beijing, China).

Amplicon sequence variants (ASV) were generated and analyzed with the Quantitative Insights into Microbial Ecology 2 (QIIME 2, version 2021.2) software program (Caporaso et al., 2010). The DADA2 algorithm was used to correct sequencing errors on primer-free reads and generate ASVs for bacterial communities within the R software suite (v3.2.5) (Callahan et al., 2016). Taxonomic identification was conducted by comparison against the 99% Greengenes clusters reference set, using the scikit-learn classifier v0.19.1 (Varoquaux et al., 2015). Collated taxonomic identification tables were created for all taxonomic levels.

Alpha diversity indices including the Chao1 and Shannon indices were calculated in QIIME 2 within the R software suite (v3.2.5). Pairwise comparisons among communities using Wilcoxon rank sum tests in R were conducted to quantitatively evaluate ASV richness variation between groups. To assess beta diversity, weighted UniFrac distances that use phylogenetic information to evaluate community similarity (Lozupone and Knight, 2007) were calculated in QIIME 2. Tables normalized with average rarefied counts were constructed to calculate Jaccard (presence/absence) and Bray–Curtis (abundance-weighted) dissimilarity matrices. Non-metric multidimensional scaling (NMDS) ordinations were generated based on the Bray-Curtis distance matrix (Vandeputte et al., 2017).

The functional potentials of the communities were predicted from the 16S rRNA gene data using PICRUSt2 (version 2.0) based on KEGG ortholog predictions (at levels 1, 2, and 3) (Langille et al., 2013). The community assembly mechanisms were estimated using normalized stochasticity (NST) algorithm package (version 3.0.3) (Ning et al., 2019) based on the weighted Bray–Curtis distances. Microbial community network analysis (Coutinho et al., 2017) was conducted for different treatment groups using the SpiecEasi package for R (v1.1.2) and using the SparCC approach (Friedman and Alm, 2012). To improve accuracy, only ASVs occurring in more than 50% of all samples were retained. The pairwise SparCC correlation coefficients between the remaining ASVs were calculated, with bootstrap *p* < 0.05 considered a valid relationship. The networks were visualized using the Gephi software package (v0.9.7) (Bastian et al., 2009).

### 2.9 Quantitative PCR analyses

Relative abundances of bacteria and Strain LVI A0039 in shrimp intestines were assessed using qPCR. To measure the abundances of strain LVI A0039, a specific gene (fragment range from 1,206,056 to 1,206,745) termed N1071 was identified in the genome that was only encoded in one copy. The two primer pairs 519F/907R (5′-CAGCMGCCGCGGTAANWC-3′/5′-CCGTCAATTCMTTTRAGTT-3′) (Zhou et al., 2022) and N1071F/N1071R (5′-TGACCCGTCGTTTACAAT-3′/5′-CTGCCTCACAAAGTC CAAG-3′) were used to estimate 16S rRNA gene abundances of bacteria overall and the N1071 gene specific to strain LVI A0039, respectively. Standard curves were generated using 10-fold serial dilutions of the plasmid pMD19-T containing the target DNA PCR fragments of the 16S rRNA and N1071 genes. Triplicate amplifications were conducted in 10 μL reaction systems containing 5 μL of a 2 × RealStar Green Fast Mixture (GenStar^®^, Beijing, China), 3 μL of sterile distilled H_2_O, 0.5 μL of each primer (10 μM), and 1.0 μL of template DNA. The thermal cycling steps were performed in a Roche Light Cycler 480 II PCR Real-Time PCR System instrument (Roche, Basel, Switzerland), including an initial denaturation step at 95°C for 5 min, then 40 cycles of 95°C for 15 s, 60°C for 20 s, and ten 72°C for 30 s. Gene copy numbers were calculated based on standard curves for the 16S rRNA and N1071 genes.

Eleven genes (*crr*, *gad*C, *yaj*C, *yid*C, *sec*A, *fts*Y, *ffh*, *inl*A, *prt*C, *lep*A, and *eta*) related to bacterial membranes were analyzed based on strain LVI A0039 genomic information. The genes were associated with three categories (cellular processes, environmental information processing, and human diseases) (Supplementary Table 2). qPCR was used to evaluate the relative abundances of these genes in shrimp intestinal communities at different times across treatment groups. Primer sequences (Supplementary Table 3) were designed using the Primer-BLAST program^7^ and generated at RuiBiotech Co., Ltd (Guangzhou, China). qPCR data were analyzed using the 2^–ΔCT^ method and normalized to *EF1*-α expression.

### 2.10 Statistical analyses

NMDS and analysis of similarity (ANOSIM) tests were performed to evaluate overall differences in bacterial communities using the Bray–Curtis distances. Analysis of variance (ANOVA) tests were conducted using SPSS 21.0 (IBM, Armonk, USA). The homogeneity of variance was evaluated with Levene’s tests before ANOVA tests. Tukey’s significant difference test was used when variances were homogeneous.

## 3 Results

### 3.1 Cultivation of CB from the intestine of *P. vanmamei*

Eleven strains (Supplementary Table 4) with 16S rRNA sequences associated with CB were successfully isolated on 1/10 NA, AB, and 1/10 2216E media. The strains were designated as LVI A0002, LVI A0005, LVI A0006, LVI A0007, LVI A0039, LVI A0062, LVI A0063, LVI A0075, LVI A0078, LVI A0079, and LVI A0080. Growth condition optimization revealed that the 11 strains grew best on the PPLO medium that was then used for subsequent strain culturing. The four strains LVI A0039, LVI A 0006, LVI A0075, and LVI A0078 exhibited the most rapid growth and the most similarity to CB based on 16S rRNA gene sequence identities. The four strains were consequently selected for further studies.

**FIGURE 1:**
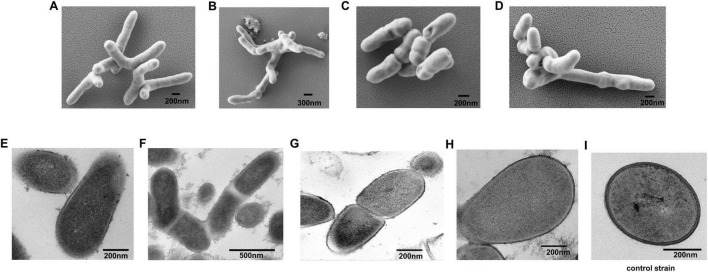
Morphology of strain A0039, A0006, A0075, and A0078. **(A–D)**. SEM observation of strain A0039, A0006, A0075, and A0078 after 3 days on PPLO agar. **(E–H)**. TEM observation of the ultrathin section of strain A0039, A0006, A0075, A0078 grown on PPLO medium for 3 days. **(I)**. TEM observation of the ultrathin section of a typical Gram-positive bacterium *Tessaracoccus* sp. A29. The arrows indicate the unit membrane.

Colonies of the four strains on PPLO medium under anaerobic conditions were small (< 1 mm diameter). The colonies exhibited round circles in the middle and rough edges, were non-pigmented, and exhibited a fried-egg appearance with microscopic observation (Supplementary Figure 2). SEM observation indicated that the cells of the four strains were rod-shaped and ramiform (Figures 1A–D). Ultrastructural examination indicated that the four strains had one single trilaminar plasma membrane and the absence of a cell wall (Figures 1E–H). By contrast, the control strain *Tessaracoccus* sp. A29 exhibited a thick peptidoglycan layer (Figure 1I).

### 3.2 Physiological and biochemical characterization

#### 3.2.1 Temperature, pH and NaCl tolerance tests

Cells of the four strains were Gram-stain-negative, anaerobic, and non-motile. The four strains exhibited different tolerances to temperature, sodium chloride (NaCl) concentrations, and pH. Strain LVI A0039 growth was observed from 25°C to 34°C, at pH of 6.0–7.0, and in the presence of up to 2% (w/v) NaCl concentrations, with optimum growth at 30–32°C, pH 7.0, and with 1% NaCl. Strain LVI A0006 grew from 25°C to 34°C, at pH of 6.0–7.0, and with NaCl ranging from 0% to 2% (w/v) with optimum growth at 30–32°C, pH 7.0, and with 1% NaCl. Strain LVI A0075 and LVI A0078 grew from 28°C to 34°C, with optimum growth at 30–32°C, and at pH 7.0–8.0. Strain LVI A0075 grew with NaCl concentrations of 2% or below, while Strain LVI A0078 grew in the presence of up to 4% NaCl (Table 1).

**TABLE 1:** Differential phenotypic characteristics of strain LVI A0006, LVI A0039, LVI A0075, LVI A0078.

	LVI A0006	LVI A0039	LVI A0075	LVI A0078
Cell morphology	rod-shaped	rod-shaped	rod-shaped	rod-shaped
pH range for growth	6–7	6–7	7–8	7–8
Temperature for growth (°C)	25–34	25–34	28–34	28–34
NaCl tolerance (% w/v)	0–3	0–2	0–2	0–4
Oxidase	–	–	–	–
Catalase	–	–	–	–
Degradation of:				
Gelatin Tween 80	w	+	w	–
Urea	–	–	–	–
Utilization of:				
α-D-Glucose	+	+	+	+
α-D-Lactose	+	w	+	+
D-Mannose	+	–	+	–
D-Raffinose	+	–	+	+
D-Mannitol	+	w	+	–
D-Sorbitol	+	–	+	+
L-Fucose	+	+	+	+
L-Rhamnose	+	+	+	+
D-Sucrose	+	w	+	+
D-Maltose	+	–	+	–
Salicin	+	–	+	+
Xylose	+	–	–	–
Arabinose	+	–	w	–
Esculin	+	+	+	+
Glycerol	+	–	w	w
D-Cellose	+	–	+	–
Melezitose	+	–	+	–

#### 3.2.2 Enzyme activities and API 20A kits tests

The four strains were negative for oxidase, catalase, and urease activities. Strain LVI A0039 exhibited positive gelatin degradation, while strains LVI A0006 and LVI A0075 only exhibited weak positive results, and Strain LVI A0078 was unable to hydrolyze gelatin. Detailed physiological and biochemical characteristics of the strains are summarized in Table 1. API 20A (Analytical Profile Index) test results indicated that Strain LVI A0039 could use α-D-glucose, L-fucose, L-rhamnose, and esculin as sole carbon sources, in addition to weakly assimilating α-D-lactose, D-mannitol, and D-sucrose. However, no assimilation of D-mannose, D-raffinose, D-sorbitol, D-maltose, salicin, xylose, arabinose, glycerol, D-cellose, or melezitose was observed. Strain LVI A0006 was able to use all sole sources of carbon within the API 20A system. Strain LVI A0075 could use α-D-glucose, α-D-lactose, D-mannose, D-raffinose, D-mannitol, D-sorbitol, L-fucose, L-rhamnose, D-sucrose, D-maltose, and salicin as sole sources of carbon, while also weakly assimilating arabinose and glycerol, but were not able to use xylose. Strain LVI A0078 was able to use α-D-glucose, α-D-lactose, D-raffinose, D-sorbitol, L-fucose, L-rhamnose, D-sucrose, salicin, and esculin as sole carbon sources, while weakly assimilating glycerol, but could not assimilate D-mannose, D-mannitol, D-maltose, xylose, arabinose, D-cellose, and melezitose.

#### 3.2.3 Fatty acid compositions of the isolated CB strains

The major fatty acids of Strain LVI A0039 included C_12:0_FAME (18.00%), C_16:0_FAME (13.89%), C_14:0_FAME (10.15%), C_18:1_CIS9FAME (10.30%), and C_16:0_DMA (9.37%). The major fatty acids composition of Strain LVI A0006 was the same as Strain LVI A0039, but with different proportions: C_12:0_FAME (19.55%) C_16:0_FAME (12.38%), C_16:0_DMA (11.37%), C_14:0_FAME (11.06%), and C_18:1_CIS9FAME (9.75%). The major fatty acid composition of Strain LVI A00075 was the same as for Strain LVI A0078, but with different abundances of C_16:0_DMA (19.88% vs 19.53%), summed feature 10 (C_18:1_c11/t9/t6FAME, 10.83% vs 16.57%), C_16:1_CIS9FAME (9.04% vs 6.05%), C_16:0_FAME (8.43% vs 6.81%), C_16:1_CIS7FAME (6.59% vs 7.17%), and C_18:1_CIS9FAME (6.46% vs 8.72%). Detailed fatty acid compositions of strains LVI A0039, LVI A0006, LVI A0075, and LVI A0078 are shown in Table 2. Based on major fatty acid compositions, Strain LVI A0039 was more closely related to Strain LVI A0006 than Strain LVI A0075 or Strain LVI A0078.

**TABLE 2:** Fatty acids components of strains LVI A0006, LVI A0039, LVI A0075 and LVI A0078.

Fatty acids components		LVI A0006	LVI A0039	LVI A0075	LVI A0078
	C10:0FAME	6.12	6.02	–	–
	iso-C_10:0_FAME	–	0.54	–	–
	C_11:0_ DMA	1.00	1.04	0.65	–
	C_12:0_ FAME	19.55	18.00	–	–
	C_14:0_ FAME	11.06	10.15	4.21	2.73
	C_14:0_ DMA	2.39	1.85	2.82	2.44
	C_14:1_-cis (n9) FAME	1.03	1.03	0.66	0.65
	C_14:1_-cis (n7) DMA	–	–	0.52	–
	C_16:0_ ALDE	1.74	1.94	4.18	4.70
	C_16:1_-cis (n7) FAME	1.76	1.94	6.59	7.17
	C_16:1_-cis (n9) FAME	1.91	2.48	9.04	6.05
	C_16:1_-cis (n11) FAME	0.84	0.99	–	0.46
	C_16:0_ FAME	12.38	13.89	8.43	6.81
	C_16:1_ cis (n9) DMA	–	–	2.06	0.86
	C_16:0_ DMA	11.37	9.37	19.88	19.53
	C_18:0_ ALDE	–	0.50	0.60	0.51
	C_18:0_ FAME	3.91	4.28	4.28	4.66
	C_18:0_ DMA	4.02	3.65	3.72	3.19
	C_18:1_-cis (n9) FAME	9.75	10.30	6.46	8.72
	C_18:1_-cis (n9) DMA	2.66	3.36	2.02	1.45
	C_18:1_-cis (n11) DMA	0.89	1.00	4.16	5.15
	C_18:2_ CIS 9,12 FAME	1.33	1.59	0.75	0.59
	C_19:0_ CYC 9,10 DMA	1.09	0.78	–	–
	19 CYC 11,12/:1FAME	–	0.27	–	–
**Summed Feature**					
	1	0.81	0.62	1.25	1.10
	4	–	–	1.75	1.34
	6	–	–	2.71	2.50
	8	–	–	1.12	1.31
	10	2.12	2.63	10.83	16.57

Summed Feature1, 4, 6, 8, 10 indicate C13:1 cis (n12) FAME, C15:2 FAME, C16:1 cis (n7) DMA, C17:1 cis(n9) FAME, C18:1c11/t9/t6 FAME.

### 3.3 Phylogenetic analysis of the isolated CB strains

**FIGURE 2:**
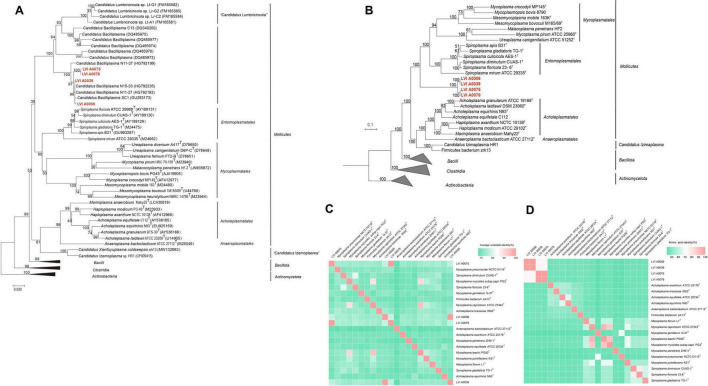
Phylogenetic analysis of Strains LVI A0006, LVI A0039, LVI A0075 and LVI A0078. **(A)**. Phylogenetic tree based on 16S rRNA gene sequences of strain LVI A0039, LVI A0006, LVI A0075, LVI A0078 and related sequences. **(B)**. Phylogenetic tree based on genome sequences. **(C)**. ANI values shared among the *Mollicutes* species. **(D)**. AAI values shared among the *Mollicutes* species. The ANI and AAI values are presented in Supplementary Table 5.

Phylogenetic trees were constructed to determine the evolutionary position of four strains in the tree of life. Pairwise comparison of the 16S rRNA gene sequences of LVI A0039, LVI A0006, LVI A0075, and LVI A0078 strains against those in the EzBioCloud database indicated that the four strains belonged to uncultured bacteria within the class *Mollicutes*, with particularly high similarity (99.25%, 99.18%, 96.58%, and 96.79% nt identity, respectively) to the accession GU293173 that was classified as a CB bacterium in the EzBioCloud and SILVA databases. The four strains were phylogenetically related to three 16S rRNA gene sequences (HG792235, HG792192, and GU293173) associated with uncultured CB and comprised a distinct evolutionary lineage (based on a neighbor-joining phylogenetic tree of 16S rRNA genes) that was independent of the other reference strains within the class *Mollicutes* (Figure 2A). The distinct clade was sister to the order *Entomoplasmatales*, to which it shared < 84.75% 16S rRNA gene sequence similarity, suggesting that the group comprising the four strains formed a new clade within the *Mollicutes*. Specifically, the clade comprising the four strains could be classified as a novel Order based on 16S rRNA gene sequence similarity and phylogenetic relationships. In addition, strains LVI A0075 and LVI A0078 shared 100.00% nt sequence similarity and formed a discrete phylogenetic group, consequently likely representing the same species. Strains LVI A0039 and LVI A0006 shared 99.45% 16S rRNA gene sequence nt similarity and clustered with another clade comprising three sequences of CB different from strains LVI A0075 and LVI A0078, suggesting they may be the same species. This relationship was also supported by Maximum Parsimony and Maximum Likelihood phylogenetic analyses (Supplementary Figure 3).

Further evidence for the affiliations of the four strains comprising a new clade of *Mollicutes* was observed with phylogenomic analyses (Figure 2B). The genomic-based analysis indicated that the four strains comprised one clade and were independent of members of different orders within the *Mollicutes* that itself was phylogenetically sister to the Orders *Entomoplasmatales* and *Acholeplasmatales*. These results confirmed that the four strains comprised a new clade within the *Mollicutes* and were a novel Order within the class. The four strains exhibited large genomic divergences based on ANI and AAI values compared with genomes of different orders within the *Mollicutes* (< 88.40% by ANIm, < 66.08% by ANIb, and < 46.67% in AAI; Figures 2C, D; Supplementary Table 5). Large divergences among the four strains and other members of the *Mollicutes* were also apparent based on DDH and tetranucleotide analyses, with < 22.30% by DDH and < 0.80 by tetranucleotide analyses (Supplementary Table 5). Strain LVI A0039 shared 99.60%, 99.33%, 99.73%, 95.60%, and 0.99 similarity with Strain LVI A0006 based on ANIm, ANIb, AAI, DDH, and tetranucleotide analyses, respectively. The genome relatedness of strains LVI A0075 and LVI A0078 based on ANIm, ANIb, AAI, DDH, and tetranucleotide analyses was 99.88%, 99.98%, 99.80%, 96.60%, and 0.99, respectively. These results suggest that strains LVI A0039 and LVI A0006 were the same species, while strains LVI A0075 and LVI A0078 were the same species.

Taken together, the morphological, physiological, biochemical, chemotaxonomic, phylogenetic, and genomic analyses indicated that the four strains comprised a novel lineage of the Class *Mollicutes*. Strain LVI A0039 is proposed as the type strain of a novel genus within the *Mollicutes*, termed *Enteroplasma penaei* gen. nov. sp. nov., with Strain LVI A0006 belonging to the same species. The phylogenetic and phylogenomic analyses indicated that the four strains comprised a clade with high bootstrap support that distinguished them from other taxa within the *Mollicutes*, consequently supporting the proposal of the Order *Enteroplasmatales* ord. nov. that includes the *Enteroplasmaceae* fam. nov. Finally, strains LVI A0075 and LVI A0078 are proposed as the novel species *Enteroplasma albus* sp. nov. Nomenclature of the four strains in Supplementary material.

### 3.4 Genomic features and functional analyses

#### 3.4.1 Genomic features of the isolated CB strains

To understand the potential function of the strain, its whole genome was sequenced. The assembly results showed that strain LVI A0039 had only one chromosome genome and no plasmid, while strain A0006 contained one chromosome genome and two plasmid genomes, and the other two strains contained one chromosome genome and one plasmid genome, respectively. The genome sizes of strains LVI A0039, LVI A0006, LVI A0075, and LVI A0078 are 2,059,165, 2,079,178, 1,907,082, and 1,965,949 bp, respectively, with G + C contents of 34.23%, 34.15%, 32.14%, and 32.11%, respectively (Supplementary Figure 4 and Supplementary Table 6). Annotation of the Strain LVI A0039 genome revealed that it comprised 1,905 coding genes and 61 ncRNA genes (46 tRNA and 15 rRNA genes). The strain LVI A0006 genome encoded 1,980 genes and 61 ncRNA genes (46 tRNA and 15 rRNA genes). The strain LVI A0075 genome encoded 1,866 genes and 64 ncRNA genes (46 tRNA and 18 rRNA genes), while the strain LVI A0078 genome encoded 1,965 genes and 61 ncRNAs (43 tRNA and 18 rRNA genes).

#### 3.4.2 Annotation of COG, KEGG and CAZy functions of the four genomes

A total of 22 COG categories (COG category C to X) were identified in the COG annotations (Supplementary Figure 5A and Supplementary Table 7). The most abundant COG categories in the genomes of strains LVI A0039 and LVI A0006 were annotated as carbohydrate transport and metabolism (COG category G, 148 and 144 genes, respectively), while the most abundant COGs in the strain LVI A0075 and LVI A0078 genomes belonged to the translation, ribosomal structure, and biogenesis category (COG category J, 140 and 140 genes, respectively).

**FIGURE 3:**
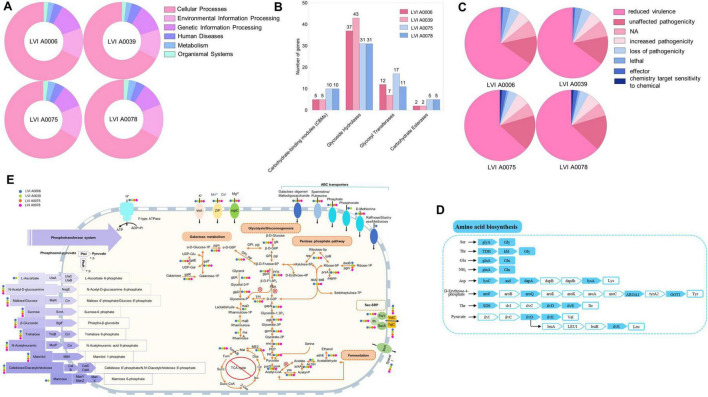
Genome analysis of the strains LVI A0006, LVI A0039, LVI A0075 and LVI A0078. **(A)**. Functional annotation of KEGG metabolic pathway genes in the genomes of four strains. **(B)**. Function annotations of CAZy database in the genomes of four strains. **(C)**. Annotations result of PHI database in the genomes of four strains. **(D)**. Amino acid biosynthesis **(E)**. Overview of metabolic capabilities of the four strains.

A total of six KEGG gene categories were annotated (cellular processes, environmental information processing, genetic information processing, human diseases, metabolism, and organismal systems) to the genomes of the four strains. The metabolism category was the most represented, followed by genetic information processing and environmental information processing (Figure 3A, Supplementary Figure 5B and Supplementary Table 8). The most abundant sub-category within the metabolism category for strains LVI A0039 and LVI A0006 was carbohydrate metabolism, with 126 genes annotated for both strains, followed by membrane transport in genetic information processing, and translation in environmental information processing genes. The most abundant metabolism sub-category for strains LVI A 0075 and LVI A0078 was also carbohydrate metabolism, comprising 98 genes annotated for both strains, followed by translation in environmental information processing, and membrane transport in genetic information processing genes. Annotation against the CAZy database revealed a total of 43, 37, 31, and 31 genes encoding glycosyl hydrolases (GH) in the strains LVI A0039, LVI A0006, LVI A0075, and LVI A0078 genomes, respectively. In addition, 7, 12, 17, and 11 genes encoded glycosyl transferases (GT) in the genomes (Figure 3B). Six phenotype genes and one unknown phenotype gene were annotated to the genomes of the four strains by comparison against the PHI database. Most of the genes were associated with the “reduced virulence” category that indicates the reduced pathogenic capacity of microorganisms (Figure 3C). Most amino acid synthesis pathway genes were missing or incomplete, except for the glutamine, threonine, and serine pathways (Figure 3D).

#### 3.4.3 Virulence factors and antibiotic-resistant genes

Whole genome sequences of the strains LVI A0006, LVI A0039, LVI A0075, and LVI A0078 were compared with the data of VFDB database to obtain information about potential virulence factors (Supplementary Tables 9, 10). The results showed that strains LVI A0006 and LVI A0039 have the same virulence genes in their genomes. There were three related sequences identified, and the gene with the highest similarity was *hys*A, with 96.6% similarity, belonged to *Streptococcus pneumoniae* TIGR4 (Supplementary Table 9). Six related sequences were identified as virulence genes in the genomes of strains LVI A0075 and LVI A0078. Among these, *neu*B exhibited the highest sequence similarity at 94.6%, categorizing it within *Staphylococcus aureus* subsp. *aureus* MW2 (Supplementary Table 10).

To detect various antibiotic resistance gene present in the four strains, the full nucleotide sequences were uploaded to CARD database for comparison, and the results were shown in Supplementary Table 11. The results showed that was only one gene associated with antibiotic resistance in the strains LVI A0006 and LVI A0039. This gene was highly matched with the *van*Y gene in glycopeptide resistance gene cluster.

#### 3.4.4 Metabolic pathway analysis

Genomic analysis indicated that the four strains could utilize sugars as a source of carbon and energy via glycolysis and the pentose phosphate pathway. Although the genomes lack the gene for the key enzyme fructose-bisphosphate aldolase that prevents the production of glyceraldehyde-3-phosphate, the pentose phosphate pathway can provide this key intermediate (Figure 3E). In addition, strains LVI A0006 and LVI A0039 can use glycerol and rhamnose to produce glyceraldehyde-3-phosphate, an intermediate product missing during glycolysis, while strains LVI A0075 and LVI A0078 can only use glycerol, but not rhamnose (Figure 3E). Pyruvate metabolism-related genes were detected in the four genomes, suggesting that pyruvate can be intracellularly metabolized to acetate or ethanol in a series of reactions involving pyruvate dehydrogenase, phosphate acetyltransferase, acetate kinase aldehyde dehydrogenase, and acetaldehyde dehydrogenase. The genes (*fum*A and *fum*B) encoding fumarate hydratase were identified in the genomes. However, none of the genomes contained complete gene sets encoding the tricarboxylic acid (TCA) cycle, rendering the TCA cycle functioning highly improbable. Given that no genes were identified encoding cytochrome C oxidase and only one gene (*SOD*2) was identified that encoded peroxisomes in the four strains, the strains are probably strictly anaerobic, consistent with the cultivation results. Many sugars, including sugar phosphotransferase system (PTS) such as maltose, glucose, trehalose, and others, are likely transported into cells via phosphoenolpyruvate. A total of 18 genes were annotated as encoding ABC transporters, suggesting that substrates such as sperimidine, putrescine, galactose oligomers, maltooligosaccharides, phosphate, phosphonate, and D-methionine could be transported into cells (Figure 3E). Five genes (*yaj*C, *yid*C, *sec*A, *ffh*, and *fts*Y) related to bacterial secretory activities were also annotated, indicating that the four strains depended on the Sec system for protein export, including adhesins and toxins. The four strains are unable to synthesize peptidoglycan due to a lack of complete peptidoglycan synthesis gene sets.

### 3.5 Verification of Strain LVI A0039 ecological function

#### 3.5.1 Colonization of Strains LVI A0039 in the intestine

**FIGURE 4:**
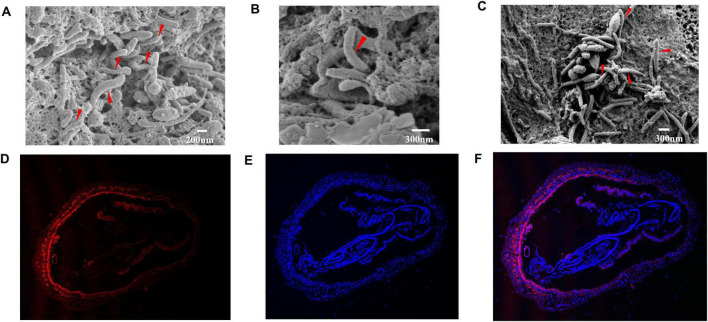
Colonization of the strain in intestine of *P. vannamei*. **(A–C)**. SEM observation of rod-shaped bacteria associated with the intestinal cuticle of *P. vannamei*. **(D–F)**. Specific detection of strain LVI A0039 associated with intestine of *P. vannamei* by in FISH, the nuclear stained by DAPI were blue under ultraviolet excitation, positive expression was red fluorescence signals labeled by cy3.

Many rod-shaped cells were observed in shrimp intestines from the RS group (reverse gavage with Strain LVI A0039) based on SEM, with bacteria exhibiting morphologies similar to Strain LVI A0039 inserted into the spaces between the intestinal microvilli or intestinal walls (Figures 4A–C, marked by red arrows). This indicated that Strain LVI A0039 may be present within shrimp intestines and may colonize intestinal walls, which was evaluated with FISH analysis. Bright red fluorescence signals were observed in the lumen, microvilli, and intestinal tissue of *P. vannamei* intestines (Figure 4D), suggesting that Strain LVI A0039 could colonize intestines and even invade shrimp intestinal tissue. DAPI counterstaining confirmed that the bacterial cells highlighted by FISH contained DNA and were thus not artifacts (Figures 4E, F).

#### 3.5.2 The analysis of 16S rRNA gene amplicon sequencing

**FIGURE 5:**
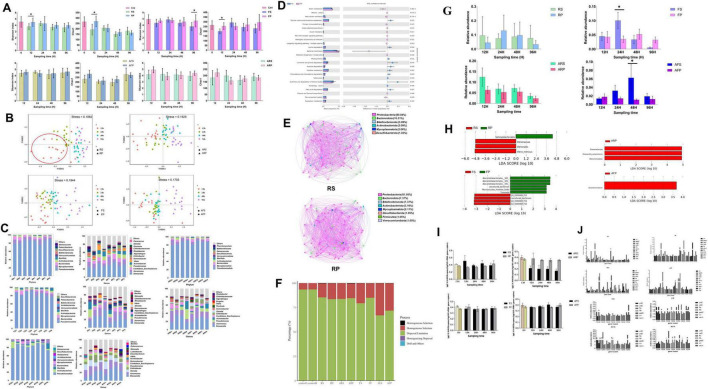
Functional analysis of strain LVI A0039 in intestine of *P. vannamei*. **(A)** and **(B)**. Represent the intestinal microbial diversity of shrimp intestine in different treatment groups, **(A)**. Stands for α-diversity analysis, **(B)**. Stands for β-diversity analysis. **(C)**. Represents the bacterial composition of shrimp intestine in different treatment groups at phyla level and genus level. **(D)**. Represents the functional prediction of RS and RP amplicon data using PICRUSt2 software. **(E)**. The inference analysis of RS and RP microbial network based on SparCC method. **(F)**. The microbial community assembly in shrimp intestines of all treatment groups. **(G)**. The relative abundance of strain LVI A0039 in different treatment groups. **(H)**. The LEfSe results of enriched of different treat groups. **(I)**. The relative abundance of strain LVI A0039 in the intestine of *P. vannamei*. **(J)**. The quantitative results of membrane genes in different treatment groups.

16S rRNA gene amplicon sequencing of shrimp intestine communities was conducted to generate additional community compositional information. A total of 1,403 prokaryotic amplicon sequence variants (ASVs) were detected in all samples of the ten groups. The ASVs were classified into 30 phyla and 412 genera. The α-diversity (as measured by Chao1 and Shannon indices) in the RS group communities was significantly lower than in the RP (reverse gavage with PBS) group at 12 h (*p* < 0.05), but not significantly different at 24 h, 48 h, and 96 h (*p* > 0.05), both of which is lower than in the control group. In the RS group, the Chao1 and Shannon indices increased at 24 h compared with 12 h, but gradually decreased at 48 h and 96 h. The Chao1 index value for the FS (fed with strain inoculum LVI A0039 mixed into feed) group was significantly lower than for the FP (fed with PBS mixed into feed) group at 12 h (*p* < 0.01), but not significantly different at 24 h, 48 h, and 96 h (*p* > 0.05). The Shannon index for the FS group was significantly lower than for the FP group at 96 h (*p* < 0.05), but significant differences were not observed at 12 h, 24 h, and 48 h (*p* > 0.05). The FS and RS groups exhibited similar trends overall, while the Chao1 and Shannon indices of the FS group indices also increased at 24 h compared with 12 h, but gradually decreased at 48 h and 96 h. The Chao1 and Shannon indices were not significantly different at 12 h, 24 h, 48 h, and 96 h in groups with antibiotics (*p* > 0.05) (ARS vs ARP; AFS vs AFP) (Figure 5A). NMDS analysis indicated that the bacterial community composition of the RS group was markedly different from that in the RP group at 12 h, but the community composition at 24 h, 48 h, and 96 h was similar between the RS and RP groups. The community compositions of the FS and FP groups were distinct at 12 h and 96 h, but similar at 24 h and 48 h. After antibiotic administration, community composition significantly changed. The community compositions of ARS and ARP groups were distinct at 12 h and 24 h, but similar at 48 h and 96 h, while the compositions of the AFS and AFP communities were significantly different at 12 h and 96 h (Figure 5B). α-diversity analyses indicated different effects after supplementation with strains by reverse gavage and mixing with feed (i.e., for the RS and FS groups). NMDS analysis suggested that the composition of IM community changed after the addition of strains.

At the phylum level, *Proteobacteria* were abundant accounting for > 70% of total communities for all groups. In the four groups without antibiotics (RS, RP, FS, and FP), the phyla *Proteobacteria*, *Mycoplasmatota*, *Bacteroidota*, *Actinobacteriota*, and *Firmicutes* were the most abundant. At the genus level, *Shewanella*, *Aeromonas*, CB, *Pseudomonas*, *Acinetobacter*, *Vibrio*, *Gemmobacter*, *Chitinibacter*, *Algoriphagus*, and *Rhodobacter* were most abundant in the RS and RP groups, while *Shewanella*, *Aeromonas*, *Pseudomonas*, *Acinetobacter*, CB, *Chitinibacter*, *Shinella*, *Gemmobacter*, *Fluviicola*, and *Vibrio* were most abundant in the FS and FP groups. After administering antibiotics, the relative abundances of the phylum *Mycoplasmatota* and the genus CB significantly decreased. In the four groups with antibiotics (ARS, ARP, AFS, and AFP), the phyla *Proteobacteria*, *Mycoplasmatota*, *Bacteroidota*, *Actinobacteriota*, and *Firmicutes* were the most abundant. At the genus level, *Shewanella*, *Aeromonas*, *Pseudomonas*, *Acinetobacter*, CB, *Chitinibacter*, *Shinella*, *Gemmobacter*, *Fluviicola*, and *Vibrio* were most abundant in the ARS and ARP groups, while *Shewanella*, *Aeromonas*, *Shinella*, *Chitinibacter*, *Pseudomonas*, CB, *Gemmobacter*, *Acinetobacter*, *Vibrio*, and *Cloacibacterium* were most abundant in the AFS and AFP groups (Figure 5C). Overall, *Proteobacteria* were especially dominant in the shrimp intestinal environment, and differences in microbial community composition were apparent under different treatments (e.g., after reverse gavage and mixing into feed).

#### 3.5.3 The analysis of microbial community function

To evaluate the metabolic functional differences between the two groups of supplemental strain LVI A0039 (RS) and without strain LVI A0039 (RP), functional potentials were predicted from the amplicon data using PICRUSt2. Several predicted pathways were significantly enriched in the RS microbiota (95% confidence intervals, *p* < 0.05). Among these pathways, genes associated with amino acid metabolism (e.g., glycine, serine, and threonine metabolism; lysine degradation; tryptophan metabolism; and histidine metabolism), lipid metabolism (fatty acid metabolism, synthesis and degradation of ketone bodies), metabolism of terpenoids and polyketides (limonene and pinene degradation), and xenobiotic biodegradation and metabolism (atrazine degradation, chloroalkane and chloroalkene degradation) were predicted to be significantly more abundant in the RS group samples than in the RP group samples (95% confidence intervals, *p* < 0.05, Figure 5D). By contrast, genes involved in cellular motility (e.g., bacterial chemotaxis) or metabolism of cofactors and vitamins (e.g., biotin metabolism) exhibited opposite trends. The functional prediction of PIRCUSt2 showed that the amino acid metabolism and lipid metabolism of intestinal microorganisms increased after supplementation of bacterial liquid. Microbial community network analysis indicated different microbial co-occurrence networks in the RS and RP groups (Figure 5E). The more keystone nodes and edges in the network, the higher the complexity of the network and the more stable the function of the microbial community. In the RS network, 97 nodes (the basic unit of participation in the network) and 857 edges (a connection between two nodes) were identified, with 95 keystone nodes also identified. Among these, 410 and 447 positive edges (positive correlation between nodes) and negative edges (positive correlation between nodes) were identified, respectively. In the RP group, 95 nodes and 1,146 edges were identified, with 52 keystone nodes including 555 and 591 positive and negative edges, respectively. The decrease of network structure complexity may be related to the decrease of microbial diversity. The results of the stochasticity or deterministic nature of community assembly showed that more than 50% belongs to dispersal limitation in all groups, which indicated that microbial community assembly in shrimp intestines of all treatment groups is dominated by stochastic processes (Figure 5F).

The relative abundances of the genus CB differed between the groups. In the RS group, the relative abundances of CB were highest at 12 h and exhibited a decreasing trend at later times. In the RP group, the relative abundances of CB were higher at 24 h than at 12 h, 48 h, and 96 h. In the FS group, the relative abundances of CB were significantly higher at 24 h than at other sampling times while also being significantly higher than in the FP group (*p* < 0.05). A similar result was observed for the RS group, where the relative abundances of CB were highest at 12 h, followed by a decreasing trend at subsequent sampling times in the ARS group. In the AFS group, the relative abundances of CB were higher at 48 h than at 12 h, 24 h, and 96 h, in addition to being significantly higher than for the AFP group (*p* < 0.05). The relative abundances of CB significantly decreased after antibiotic treatment, indirectly indicating that increased CB abundances in groups with antibiotics (ARS and AFS) were due to supplementation of Strain LVI A0039. Variation in CB relative abundance was also apparent among groups (Figure 5G).

Linear discriminant analysis Effect Size (LEfSe) analysis indicated that the presence of specific phylotypes were differentially abundant among groups. Changes in the relative abundances of four ASVs in the RS and RP groups were the primary contributors to observed diversity differences. One ASV was assigned to the order *Sphingobacteriales* and enriched in the RP group, while three ASVs were enriched in the RS group that were classified to the order *Virbrionales*, the family *Vibrionaceae*, and *Vibrio minicus*. Changes in the relative abundances of ten ASVs were the main contributors to the observed diversity differences between the FS and FP groups, of which four ASVs were enriched in the FS group and the other six ASVs were abundant in the FP group. Three ASVs were responsible for differences between the ARS and ARP groups and enriched in the ARP group. Notably, all three ASVs were assigned to the order *Alteromonadales*, and one of these was assigned to the family *Shewanellaceae*, while the other one was assigned to the species *Shewanella amazonensis*. One ASV was more abundant in the AFP group and classified to the genus *Aurantimicrobium* (Figure 5H). *Vibrionales* members were enriched in the RS group, indicating that CB supplementation result in the increased relative abundances of *Vibrionales*.

#### 3.5.4 Changes in relative abundance of strain LVI A0039 and genes related to membrane

The relative abundance of Strain LVI A0039 in shrimp intestines after supplementation was measured with Quantitative PCR (qPCR). The relative abundance of Strain LVI A0039 in the RS group individuals increased at 12 h compared with the control, but their relative abundances gradually decreased during subsequent experiments. The relative abundance of Strain LVI A0039 in the RS group was higher than in the RP group at 12 h, 24 h, and 48 h. The relative abundance of Strain LVI A0039 in the FS group was lower than in the FP group at 12 h, but no differences were observed in the FS and FP groups at 24 h, 48 h, and 96 h. The relative abundance of LVI A0039 gradually decreased in the ARS group during the experiment and was lower than in the ARP group. The relative abundance of strain LVI A0039 in the AFS group gradually increased between 12 h and 48 h and was higher than in the AFP group but decreased at 96 h and was lower than in the AFP group. qPCR analysis of Strain LVI A0039 in shrimp intestines indicated that reverse gavage is a relatively effective mechanism to supplement the strain, with the relative abundance of Strain LVI A0039 altered in the reverse gavage groups (RS and ARS), but not in the feed mixing groups (FS and AFS) (Figure 5I).

qPCR analyses further revealed that the relative abundances of 11 genes related to bacterial membranes (*crr*, *gad*C, *yaj*C, *yid*C, *sec*A, *fts*Y, *ffh*, *inl*A, *prt*C, *lep*A, and *eta*) were different in eight groups at different sampling times (Figure 5J). In the RS group, the relative abundances of *crr*, *gad*C, *yaj*C, *ffh*, and *eta* at 12 h were higher than at 24 h, 48 h, and 96 h. In the RP group, the relative abundances of *gad*C, *yaj*C, *lep*A, and *inl*A at 96 h were higher than at 12 h, 24 h, and 48 h, while *ffh, prt*C, and *eta* abundances at 48 h were higher than at 12 h, 24 h, and 96 h. In the ARS group, the relative abundances of *yid*C and *ffh* at 12 h and 24 h were higher than at 48 h and 96 h. In the ARP group, the relative abundances of *yid*C at 24 h were higher than at 12 h, 48 h, and 96 h, but were lower than in the ARS group. In the FS group, the relative abundances of *sec*A at 48 h were higher than at 12 h, 24 h, and 96 h. In the FP group, the relative abundances of *yid*C, *gad*C, and *sec*A at 12 h and 48 h were higher than at other times, although the relative abundances of *ffh* and *crr* at 12 h were higher. For the AFS group, the relative abundances of *yaj*C at 24 h were higher than that at 12 h, 48 h, and 96 h. In the AFP group, the relative abundances of *fts* at 48 h were higher than at 12 h, 24 h, and 96 h. Variation in the relative abundances of *yaj*C, *crr*, and *eta* in the RS group was the same as for CB, indicating that the addition of Strain LVI A0039 might cause variation in the abundances of these genes (Figure 5J).

## 4 Discussion

Increasing numbers of studies emphasize the role of gut symbionts in the health and disease of hosts (Fukuda et al., 2011; Littman and Pamer, 2011). An estimated ∼25% of microorganisms belong to phyla with uncultured relatives that may have important roles in ecosystem functioning (Lloyd et al., 2018). CB bacteria are widely distributed in arthropod intestines and may be involved in host physiological processes or may even affect host health (Kostanjsek et al., 2007; Hou et al., 2018). However, pure CB bacteria cultures are not available, hindering the accurate determination of their physiological traits. In this study, the first CB bacterial representatives were isolated and subjected to a detailed exploration of their physiology, genomic traits, and phylogenetic histories. In addition, a model was proposed of their central metabolic pathways, and their effects on the functioning of the intestinal bacterial community of *P. vanmamei* were explored. Overall, the study elucidated the potential mechanisms of CB that affect the intestinal health of shrimp and provided new evidence for evaluating relationships between intestinal symbiotic bacteria and their hosts.

### 4.1 Various methods contribute to culture-dependent of uncultured bacteria

Microorganisms are often difficult to culture under laboratory conditions because they may be dormant, viable but nonculturable (VBNC) (Mu et al., 2018), have low abundances in natural environments or depend on specific environmental conditions and/or the presence of various growth factors such as vitamins, amino acids, nucleotides, inorganic compounds, humic acids, or other external electron shuttles (Lewis et al., 2021). Consequently, different isolation strategies have been used for various uncultured microorganisms. To culture dominant active bacteria, simulating natural environments combined with diluting a sample “to extinction” (Rappe et al., 2002) would theoretically be able to bring abundant species into the culture. For example, the isolation and culturing of the SAR11 clade (*Pelagibacterales*) (Rappe et al., 2002) used this approach. In the present study, diluting samples 100- and 1,000-fold yielded more isolates, including CB strains when diluting the samples 1,000-fold, suggesting the success of the dilution-to-extinction method for microbial isolation (Stingl et al., 2007). To culture low-abundance bacteria, tailoring various synthetic media to culture bacteria that are adapted to specific environmental niches is an effective strategy. Culturomics has developed from the above principles and has achieved considerable success in culturing previously uncultured members of the human gut microbiota (Lagier et al., 2018). Su et al. (2018) enhanced the cellulose-degrading capacity of bacterial communities in composting using the resuscitation-promoting factor (Rpf) protein to resuscitate VBNC bacteria. In addition to the above, generating MAGs from metagenomic datasets using computational bioinformatic tools to predict the metabolic potential of strains is an effective method to isolate uncultured microorganisms. *Candidatus* Izemoplasma bacteria were reported to degrade extracellular DNA by genomic analysis, with strains subsequently successfully isolated by adding *Escherichia coli* DNA to cultures during enrichment (Zheng et al., 2021). Although CB bacteria are widely distributed in arthropods, their MAGs have not been generated in previous studies, leading to an inability to predict their metabolic potential. Consequently, the use of additional media types is the most appropriate method to culture these organisms based on their natural environments. First, cultivation methods of related strains were identified using the KOMODO platform that utilizes 16S rDNA sequences or NCBI taxon IDs to generate predictions (Oberhardt et al., 2015). Second, various types of media are used to culture sample dilutions. Previous studies have demonstrated that most microorganisms are in oligotrophic states, and thus, greater microbial isolate diversity can be obtained using an oligotrophic medium (Pulschen et al., 2017). Third, considering that CB bacteria exist in intestines of shrimp, they were hypothesized to be anaerobic, leading to the incubation of samples anaerobically. CB bacteria were ultimately successfully isolated using these methods, revealing that CB grew better on the PPLO medium during culture optimization, confirming that culture methods used for similar groups may be effective when limited knowledge of target organisms is available.

### 4.2 Culture characteristics and taxonomy of the isolated CB strains

CB bacteria have been proposed to comprise a candidate genus (Kostanjsek et al., 2007) that has not been validly published due to the lack of pure culture strains or MAGs. In this study, an efficient culture method was developed to successfully obtain four strains (LVI A0039, LVI A0006, LVI A0075, and LVI A0078) related to *Candidatus* Bacilliplasma. Transmission electron microscopy suggested that the four strains only have a plasma membrane, but no peptidoglycan layer cell wall, while colonies on PPLO solid media exhibit a fried-egg appearance that is the most prominent morphological feature of *Mollicutes* class members, consistent with the phylogenetic affiliation of the strains with *Mollicutes* (Daniel et al., 2010). Most cultivable species of *Mollicutes* require special growth factors such as sterols and fetal bovine serum (Hackett and Whitcomb, 1995), although the four strains identified in this study grew well in PPLO media without serum. Molecular analysis of the four strains indicated that the strains shared < 86% 16S rRNA gene sequence identity to the closest related type stain (*Spiroplasma floricola* 23-6^T^), which is below the threshold used to differentiate orders (86%–89%). Furthermore, ANI comparisons between the four strains and the closest related type stain were < 84%, which is well below the commonly used ANI criterion for species identification (95%–96%) (Richter and Rosselló-Móra, 2009). AAI analysis revealed AAI values between the four strains and the closest related type stain as < 46%, indicative of affiliation with a different family (based on a ∼45%–65% threshold) (Konstantinidis et al., 2017). Nevertheless, the 16S rRNA gene and AAI/ANI value thresholds proposed for defining taxonomic ranks are approximate, and phylogenetic analyses should be used to confirm taxonomic differentiation. Phylogenetic analyses based on 16S rRNA genes revealed that the four strains comprise a novel lineage along with other uncultured bacteria in the class *Mollicutes*, are well-differentiated from the four validly published orders of the class *Mollicutes* (Freundt et al., 1984; Robinson and Freundt, 1987; Tully et al., 1993; Freundt, 1955), and are most closely related to the order *Entomoplasmatales*. Genomic analyses indicated that the four strains comprised a single branch between the orders *Entomoplasmatales* and *Acholeplasmatales*. Thus, the four strains comprise a new clade within the class *Mollicutes* and likely belong to a novel order within the *Mollicutes*, leading to the proposal of *Enteroplasmatales* ord. nov. that includes the *Enteroplasmaceae* fam. nov. Based on the widely accepted thresholds of species designation as 16S rRNA gene similarity < 98.7% (Chun et al., 2018), ANI values < 95% (Richter and Rosselló-Móra, 2009), and AAI values < 95% (Konstantinidis et al., 2017), the four strains could be divided into two proposed species including *Enteroplasma penaei* gen. nov. sp. nov. and *Enteroplasma albus* sp. nov.

### 4.3 Unique genomic characteristics of *Mollicutes*

Members of the class *Mollicutes* are some of the smallest known self-replicating organisms (Halbedel et al., 2007). Their genomes are strongly reduced in size and so are their metabolic capabilities (Robinson et al., 1975). Accordingly, the four strains of this study have truncated respiratory systems, lacked a complete tricarboxylic acid cycle and quinones or cytochromes, precluding a capacity to conduct oxidative phosphorylation (Rattner et al., 2021). Culture experiments confirmed that CB are strictly anaerobic, with only minimal levels of ATP that could be generated through glycolysis. The presence of *SOD2* genes in their genomes may facilitate the survival of these bacteria during oxidative stress. Although *E. penaei* and *E. albus* are closely phylogenetically related to *Spiroplasma* species, *Spiroplasma* are facultative anaerobic, and their genomes contain complete gene sets enabling oxidation of pyruvate to acetyl-coA (Cocks et al., 1985), contrasting with *E. penaei* and *E. albus*. Despite the absence of *FBA* in their genomes, glyceraldehyde-3-phosphate can be produced via the pentose phosphate pathway or hydrolysis of glycerol, allowing glycolysis to proceed, as identified in other *Mollicutes* (Cocks et al., 1985). Genes enabling the use of glycerol are present in all *Mycoplasma* species with the exception of *M. synoviae* and are not encoded in the genomes of *Ureaplasma*, *Phytoplasma*, and *Mesoplasma* species (Robinson et al., 1975). Many genes were identified that were involved in transport functions, including phosphoenolpyruvate phosphotransferase systems (PTS) and ATP binding cassette (ABC) transporters, like that observed for the genomes of *Spiroplasma* and *Entomoplasma* genera (Daniel et al., 2010). Genomic analysis indicated that the four strains were unable to synthesize peptidoglycan and therefore could not form cell walls, consistent with one of the distinctive features of *Mycoplasmatota* that separate them from *Firmicutes* (Zheng et al., 2021). Intriguingly, many biosynthetic pathways (e.g., those for essential amino acids, vitamins, and cofactors) are partial in the four strain genomes, suggesting that they may rely on their hosts or other bacteria in the shrimp intestines for these nutrients (Lo et al., 2016).

### 4.4 CB bacteria play important roles in the intestine of arthropods

Symbiotic associations of *Mollicutes* in arthropod intestinal tracts are common and beneficial for hosts (Leclercq et al., 2014). The *Candidatus* Hepatoplasma crinochetorum is thought to improve the survival of the host under low nutritional conditions (Fraune and Zimmer, 2008). As symbiotic bacteria, spiroplasmas can mutually live with their hosts, protecting *Drosophila* and aphids from parasitic wasps, nematodes and fungal pathogens (Xie et al., 2010; Cockburn et al., 2013; Łukasik et al., 2013). However, spiroplasmas found in other hosts have been identified as pathogens, such as *P. vannamei* (Nunan et al., 2004), *bees* (Clark, 1977), *mosquitoes* (Phillips and Humphery-Smith, 1995). Studies have shown that the pathogenic mechanism of spiroplasma lies in the ability of the bacteria to cross the intestinal barrier, and the bacteria infect other tissues of the host through hemolymph (Kwon et al., 1999; Nunan et al., 2005). CB were the symbiotic bacteria that colonize the cuticular surface of terrestrial isopod hindguts, and recent studies have confirmed that CB bacteria are kind of bacteria widely existing in the intestinal tract of aquatic animals (Kostanjsek et al., 2007; Wang et al., 2021; Chen et al., 2015), CB bacteria were the indigenous populations of Chinese mitten crab, can maintain a high abundance even when the host is hungry (Wang C. H. et al., 2019). The relative abundance of CB bacteria in the gut of Chinese mitten crab with hepatopancreatic necrosis is significantly lower than that of healthy Chinese mitten crab (Zhan et al., 2022). However, the relative abundance of CB in *P. vannamei* with WFS is higher than that in healthy *P. vannamei*, which is an indicator species of WFS (Hou et al., 2018). In this study, FISH and SEM analyses in this study indicated that strain LVI A0039 is a commensal bacterium in *P. vanmamei* intestines that colonizes intestinal tissue, while no abnormal death was found during the experiment. In addition, Strain LVI A0039 is a strict anaerobic bacterium and will not survive if it can cross the intestinal barrier and enter the hemolymph, so there is no pathogenic mechanism of spiroplasmas-the pathogenicity of spiroplasmas is related to the ability of bacteria to cross the intestinal cutis barrier (Nunan et al., 2005), and the existence of CB bacteria has not been found in hemolymph. In the RS and ARS groups, the relative abundances of strain LVI A0039 first increased and then decreased during the experiment, with their relative abundances peaking at 24 h, then decreasing to the same level as before supplementation with the strain inoculum. CB bacteria were considered as indicator species in the intestines of shrimp with white feces syndrome (WFS) in previous studies (Hou et al., 2018). However, genomic analysis results showed that the virulence of the strain was weakened, and no symptoms of WFS were found after the addition of CB strains in shrimp during experiment. These results indicate that shrimp intestinal microbiota maintain a relatively balanced state, enabling host health and thereby indicating that Strain LVI A0039 is not a pathogenic bacterium. The *yidC* gene encodes a membrane-anchored periplasmic protein that plays an important role in the symbiosis between bacteria and host by helping bacteria colonize host intestines and was identified in the strain LVI A0039 genome (Nguyen et al., 2014; Pogoreutz et al., 2022). Analysis of 16S rRNA gene amplicon data indicated significantly higher relative abundances of strain LVI A0039 in the RS and ARS groups at 12 h compared with the RP and ARP groups. Furthermore, *yidC* gene copies were higher in the RS and ARS groups compared with the RP and ARP groups, indirectly confirming that strain LVI A0039 colonized shrimp intestines. Genomic analysis further revealed that strain LVI A0039 lacked complete pathways to synthesize amino acids, vitamins, and coenzyme factors, suggesting that it requires symbiosis with the host or other bacteria to obtain these nutrients (Castresana, 2000).

In summary, CB members are symbiotic bacteria widely found in arthropods, which can attach to intestinal microvilli or even enter intestinal tissues (Kostanjsek et al., 2007; Hou et al., 2018; Chen et al., 2015). It cannot be simply defined as beneficial or harmful to the host, and the changes of its abundance may make it play different functions in the host intestine. This study enriched the intestinal microbial resources of shrimp, improved the understanding of the important role of uncultured symbiotic bacteria in host health, and initially revealed the microecological mechanism of the host-symbiont interaction, providing theoretical basis and technical support for the microecological prevention and control of shrimp culture health and diseases. However, we have not confirmed what causes CB bacteria to play a double-edged sword function in the intestine of *P. vanmamei*, is it a change in relative abundance of CB bacteria or the production of certain metabolites. Correspondingly, our future endeavors would be to compare the effects of different doses of CB strains on the growth and intestinal microbial community function of *P. vanmamei*, as well as the characteristics of intestinal metabolomics.

## 5 Conclusion

Here, four strains of an uncultured bacterial group were successfully isolated from shrimp intestines and comprised members of a proposed novel order, novel family, novel genus, and two novel species. Investigation of the phylogenetic diversity, whole genomes, and cultivation-based characteristics of the four strains indicated that they were symbiotic bacteria. These results provide a framework for future research into their relative impacts on host health. Further studies will help elucidate the microecological roles of the strains and their interrelationships with hosts or other bacteria.

## Data Availability

The datasets presented in this study can be found in online repositories. The names of the repository/repositories and accession number(s) can be found in the article/Supplementary material.

## Footnotes

1
https://www.bacterio.net/

2
http://jspecies.ribohost.com/jspeciesws/

3
http://cloud.majorbio.com/page/tools/

4
http://ggdc.dsmz.de/

5
https://www.ezbiocloud.net

6
https://github.com/nylander/catfasta2phyml

7
https://www.ncbi.nlm.nih.gov/tools/primer-blast/
