# Supplementary material for: Succeed to culture a novel lineage symbiotic bacterium of Mollicutes which widely found in arthropods intestine uncovers the potential double-edged sword ecological function
Source: Front Microbiol. 2024 Oct 18;15:1458382. doi: 10.3389/fmicb.2024.1458382 (PMC11527720; doi:10.3389/fmicb.2024.1458382)
Supplement: Supplementary file 1 [file Data_Sheet_1.docx]

**Supplementary Materials**

**This supplementary materials file contains the following:**

Supplementary Tables 1 to 11

Supplementary Figures 1 to 5

Taxonomic description of novel members in the Class *Mollicutes.*

**Supplementary Tables**

**Table S1** The detailed grouping information of for verification of strain ecological functions

| Groups | Designation | Description |
| --- | --- | --- |
| I | RS | reverse gavage with strain LVI A0039 |
| II | RP | reverse gavage with PBS |
| III | ARS | reverse gavage with strain LVI A0039 after antibiotic treatment |
| IV | ARP | reverse gavage with PBS after antibiotic treatment |
| V | FS | fed with strain inoculum LVI A0039 mixed into feed |
| VI | FP | fed with PBS mixed into feed |
| VII | AFS | fed with strain inoculum LVI A0039 mixed into feed after antibiotic treatment |
| VIII | AFP | fed with PBS mixed into feed after antibiotic treatment |

R indicates reverse gavage, S indicates strain LVI A0039, P indicates PBS solution, A indicates antibiotic treatment, F indicates mixed into feed.

**Table S2** The information of 11 genes related to bacterial membranes were analyzed based on strain LVI A0039 genome.

| Gene name | Pathway Level1 |
| --- | --- |
| crr | Cellular Processes |
| gadC | Cellular Processes |
| yajC | Environmental Information Processing |
| yidC | Environmental Information Processing |
| secA | Environmental Information Processing |
| ftsY | Environmental Information Processing |
| ffh | Environmental Information Processing |
| inlA | Human Diseases |
| prtC | Human Diseases |
| eta | Human Diseases |
| lepA | Human Diseases |

**Table S3** Primers used for qRT-PCR.

| Primer name | Nucleotide Sequence (5’-3’) |
| --- | --- |
| 519-F | CAGCMGCCGCGGTAANWC |
| 907-R | CCGTCAATTCMTTTRAGTT |
| N1071-F | TGACCCGTCGTTTACAAT |
| N1071-R | CTGCCTCACAAAGTC CAAG |
| crr-F | ACGGTTTCGCTATTGAGCCA |
| crr-R | TTGCGTGACCAGTAGCTTCG |
| gadC-F | GTGTGGCGTGGTACTACTGG |
| gadC-R | ATCGAGTTCGCTTCGAGTCC |
| yajC-F | GATCGCGTTCGTTCAGTTGG |
| yajC-R | ATGTCACCAGATCCGCTACG |
| yidC-F | TCAGCACTGTACAAGCGTCA |
| yidC-R | AGCAGTCCACGAATTGCATCA |
| secA-F | CGATGTTGTAAACCCTGCCG |
| secA-R | CCTTGACGTCCACTACGACC |
| ftsY-F | ATCTACGATGCGATGGACCG |
| ftsY-R | ACCATCAGGTCGGCTTTGTT |
| ffh-F | ACGCTTGGTAAACAGCTTGC |
| ffh-R | AATCGTGACCGTGCTCCTTT |
| inlA-F | AATGTGCGTAGGTAATGCCC |
| inlA-R | TTGCGATACCAGCAAGCCA |
| prtC-F | ATACGCTGGGTACGTTCTCG |
| prtC-R | TCCAAGGTGTCACCGACTTT |
| eta-F | GTGACAGCAACGCTTTATGCT |
| eta-R | ATTGCGATACCAGCAAGCCA |
| lepA-F | AATCGATACACCGGGACACG |
| lepA-R | GCTTCGATTCCTTGAGCTGC |
| EF1-α-F | CCTATGTGCGTGGAGACCTTC |
| EF1-α-R | GCCAGATTGATCCTTCTTGTTGAC |

**Table S4** The isolation information of strains for genus *Candidatus* Bacilliplasma

| Strain Number | Medium | Accession number of sequence | Similarity (%) | Taxonomy（Based on Silva database） |
| --- | --- | --- | --- | --- |
| LVI A0002 | AB | HG792192 | 99.27 | Uncultured bacterium, *Candidatus* Bacilliplasma |
| LVI A0005 | 1/10NA | HG792192 | 98.67 | Uncultured bacterium, *Candidatus* Bacilliplasma |
| LVI A0006 | 1/10NA | HG792192 | 99.47 | Uncultured bacterium, *Candidatus* Bacilliplasma |
| LVI A0007 | AB | HG792192 | 99.34 | Uncultured bacterium, *Candidatus* Bacilliplasma |
| LVI A0039 | AB | HG792192 | 99.54 | Uncultured bacterium, *Candidatus* Bacilliplasma |
| LVI A0062 | 1/102216E | HG792192 | 99.21 | Uncultured bacterium, *Candidatus* Bacilliplasma |
| LVI A0063 | AB | HG792235 | 97.55 | Uncultured bacterium, *Candidatus* Bacilliplasma |
| LVI A0075 | 1/10 2216E | HG792235 | 97.50 | Uncultured bacterium, *Candidatus* Bacilliplasma |
| LVI A0078 | 1/10 NA | HG792235 | 97.44 | Uncultured bacterium, *Candidatus* Bacilliplasma |
| LVI A0079 | 1/10 AB | HG792235 | 97.50 | Uncultured bacterium, *Candidatus* Bacilliplasma |
| LVI A0080 | 1/10 NA | HG792235 | 97.54 | Uncultured bacterium, *Candidatus* Bacilliplasma |

**Table S5** Genome relatedness values of the four strains in this study and other 17 strains of class *Mollicutes*.

| Genome | ANIm | ANIb | DDH | AAI | Tetra |
| --- | --- | --- | --- | --- | --- |
| LVI A0039 | 1 | 1 | 1 | 1 | 1 |
| LVI A0006 | 99.60 | 99.33 | 95.60 | 99.73 | 0.99889 |
| LVI A0075 | 84.94 | 74.40 | 20.50 | 73.60 | 0.93336 |
| LVI A0078 | 84.92 | 74.39 | 20.60 | 73.58 | 0.93267 |
| *Mesoplasma florum* L1^T^ (CP006778) | 84.78 | 65.70 | 21.10 | 46.53 | 0.77327 |
| *Acholeplasma equifetale* ATCC 29724^T^ (JHXL00000000) | 88.41 | 64.99 | 19.60 | 46.67 | 0.7243 |
| *Mycoplasma putrefaciens* (CP003021) | 84.23 | 66.08 | 21.00 | 45.60 | 0.74225 |
| *Spiroplasma diminutum* (CP005076) | 84.13 | 65.27 | 22.30 | 45.44 | 0.75372 |
| *Spiroplasma gladiatoris* (CP038013) | 84.01 | 65.32 | 21.90 | 45.74 | 0.73376 |
| Firmicutes bacterium zrk13 (CP048914) | 83.65 | 64.44 | 18.70 | 46.88 | 0.68577 |
| *Spiroplasma floricola* (CP025057) | 83.62 | 65.15 | 22.30 | 45.44 | 0.7332 |
| *Mycoplasma mycoides* (ANIV01000000) | 83.62 | 65.05 | 21.20 | 45.61 | 0.70993 |
| *Mycoplasma capricolum subsp. capricolum* ATCC 27343^T^ (CP000123) | 83.60 | 65.52 | 21.30 | 45.71 | 0.74741 |
| *Acholeplasma brassicae* (FO681348) | 83.59 | 64.27 | 20.50 | 46.27 | 0.43009 |
| *Mycoplasma leachii* (CP002108) | 83.59 | 65.10 | 21.30 | 45.66 | 0.73449 |
| *Acholeplasma axanthum* (AUAL00000000) | 83.34 | 64.48 | 21.60 | 45.80 | 0.82428 |
| *Mycoplasma genitalium* (CP003773) | 82.34 | 65.85 | 16.90 | 43.29 | 0.56049 |
| *Acholeplasma equirhinis* (JAFIDC000000000) | 82.27 | 64.59 | 19.70 | 46.00 | 0.68138 |
| *Mycoplasma pneumoniae* (CP010546) | 82.07 | 65.95 | 17.00 | 43.49 | 0.49493 |
| *Mycoplasma penetrans* (RCHY01000000) | 80.59 | 65.60 | 17.70 | 43.82 | 0.79806 |
| *Anaeroplasma bactoclasticum* (QXEV00000000) | 0 | 62.86 | 0 | 46.35 | 0.50757 |

| Features | LVI A0006 | LVI A0039 | LVI A0075 | LVI A0078 |
| --- | --- | --- | --- | --- |
| Gene Bank ID | CP136989-CP136991 | CP136953 | CP136954-CP136955 | CP136956-CP136957 |
| Genome size(bp) | 2,079,178 | 2,059,165 | 1,907,082 | 1,965,949 |
| GC content (%) | 34.15 | 34.23 | 32.14 | 32.11 |
| No. contigs | 3 | 1 | 2 | 2 |
| No. plasmids | 2 | 0 | 1 | 1 |
| No. of genes | 1,980 | 1,905 | 1,886 | 1,965 |
| No. of RNAs |  |  |  |  |
| 5S rRNA | 5 | 5 | 6 | 6 |
| 16S rRNA | 5 | 5 | 6 | 6 |
| 23S rRNA | 5 | 5 | 6 | 6 |
| tRNAs | 46 | 46 | 46 | 43 |

**Table S6** Genomic features of the strains LVI A0006, LVI A0039, LVI A0075 and LVI A0078.

**Table S7** The COG annotations result of genome of four strains

| Functional classification | Description | Number of genes | | | |
| --- | --- | --- | --- | --- | --- |
|  |  | LVI A0006 | LVI A0039 | LVI A0075 | LVI A0078 |
| C | Energy production and conversion | 38 | 38 | 38 | 38 |
| D | Cell cycle control, cell division, chromosome partitioning | 5 | 5 | 7 | 7 |
| E | Amino acid transport and metabolism | 68 | 66 | 63 | 64 |
| F | Nucleotide transport and metabolism | 54 | 54 | 57 | 57 |
| G | Carbohydrate transport and metabolism | 144 | 148 | 96 | 96 |
| H | Coenzyme transport and metabolism | 35 | 35 | 33 | 33 |
| I | Lipid transport and metabolism | 34 | 34 | 29 | 29 |
| J | Translation, ribosomal structure and biogenesis | 141 | 141 | 140 | 140 |
| K | Transcription | 50 | 42 | 38 | 41 |
| L | Replication, recombination and repair | 40 | 41 | 42 | 43 |
| M | Cell wall/membrane/envelope biogenesis | 41 | 41 | 35 | 35 |
| N | Cell motility | 6 | 5 | 7 | 8 |
| O | Posttranslational modification, protein turnover, chaperones | 35 | 35 | 33 | 33 |
| P | Inorganic ion transport and metabolism | 39 | 38 | 43 | 43 |
| Q | Secondary metabolites biosynthesis, transport and catabolism | 8 | 8 | 9 | 9 |
| R | General function prediction only | 52 | 52 | 56 | 56 |
| S | Function unknown | 24 | 25 | 30 | 31 |
| T | Signal transduction mechanisms | 19 | 19 | 24 | 24 |
| U | Intracellular trafficking, secretion, and vesicular transport | 7 | 8 | 7 | 7 |
| V | Defense mechanisms | 19 | 19 | 16 | 16 |
| W | Extracellular structures | 1 | 1 | 1 | 1 |
| X | Mobilome: prophages, transposons | 2 | 2 | 4 | 4 |

**Table S8** KEGG pathway annotation in the genomes of four strains

| Functional classification | Description | Number of genes | | | |
| --- | --- | --- | --- | --- | --- |
|  |  | LVI A0006 | LVI A0039 | LVI A0075 | LVI A0078 |
| Cellular Processes | Transport and catabolism | 1 | 1 | 1 | 1 |
|  | Cellular communty-prokaryotes | 14 | 14 | 17 | 17 |
|  | Cell growth and death | 7 | 7 | 7 | 7 |
| Environmental Information  Processing | Membrane transport | 93 | 92 | 71 | 71 |
|  | Translation | 71 | 71 | 72 | 72 |
|  | Transcription | 3 | 3 | 3 | 3 |
|  | Replication and repair | 20 | 20 | 19 | 19 |
|  | Folding, sorting and degradation | 19 | 19 | 20 | 20 |
| Human Disease | Neurodegenerative diseases | 2 | 2 | 2 | 2 |
|  | Infectious diseases: Viral | 1 | 1 | 1 | 1 |
|  | Infectious diseases: Bacterial | 13 | 6 | 5 | 6 |
|  | Endocrine and metabolic diseases | 3 | 3 | 3 | 3 |
|  | Drug resistance: Antineoplastic | 4 | 4 | 5 | 5 |
|  | Drug resistance: Antimicrobial | 9 | 9 | 7 | 7 |
|  | Cardiovascular | 2 | 2 | 2 | 2 |
|  | Cancers: Specific types | 1 | 1 | 1 | 1 |
|  | Cancers: Overview | 5 | 5 | 5 | 5 |
| Metabolism | Xenobiotics biodegradation and metabolism | 14 | 14 | 14 | 14 |
|  | Nucleotide metabolism | 56 | 56 | 59 | 59 |
|  | Metabolism of terpenoids and polyketides | 12 | 12 | 9 | 9 |
|  | Metabolism of other amino acids | 17 | 17 | 17 | 17 |
|  | Metabolism of cofactors and vitamins | 34 | 34 | 35 | 35 |
|  | Lipid metabolism | 25 | 25 | 22 | 22 |
|  | Glycan biosynthesis and metabolism | 10 | 11 | 8 | 8 |
|  | Global and overview maps | 219 | 218 | 202 | 202 |
|  | Energy metabolism | 39 | 39 | 40 | 40 |
|  | Carbohydrate metabolism | 126 | 126 | 98 | 98 |
|  | Biosynthesis of other secondary metabolites | 14 | 14 | 10 | 10 |
|  | Amino acid metabolism | 40 | 40 | 34 | 34 |
| Organismal Systems | Nervous system | 1 | 1 | 1 | 1 |
|  | Immune system | 2 | 2 | 3 | 3 |
|  | Environmental adaptation | 2 | 2 | 2 | 2 |
|  | Endocrine system | 7 | 7 | 7 | 7 |
|  | Aging | 4 | 4 | 4 | 4 |

**Table S9 Virulence factor-related coding genes of strains LVI A0006 and LVI A0039**

| VFDB internal ID | Virulence gene name | Belong to the strain | Length | Similarity degree (%) |
| --- | --- | --- | --- | --- |
| >VFG001364 | *hysA*, hyaluronate lyase | *Streptococcus pneumoniae* TIGR4 | 3201 | 96.6% (28/29) |
| >VFG000079 | *clpC*, endopeptidase Clp ATP-binding chain C | *Listeria monocytogenes* EGD-e | 2463 | 86.8% (59/68) |
| >VFG002409 | *essC*, type VII secretion system protein EssC, FtsK/SpoIIIE family ATPase | *Staphylococcus aureus* subsp. *aureus* MW2 | 4440 | 84.9% (62/73) |

**Table S10 Virulence factor-related coding genes of strains LVI A0006 and LVI A0039**

| VFDB internal ID | Virulence gene name | Belong to the strain | Length | Similarity degree (%) |
| --- | --- | --- | --- | --- |
| >VFG001338 | neuB, N-acetyl neuramic acid synthetase NeuB | *Streptococcus agalactiae* 2603V/R | 1026 | 94.6% (35/37) |
| >VFG001280 | sdrD, Ser-Asp rich fibrinogen-binding bone sialoprotein-binding protein | *Staphylococcus aureus* subsp. *aureus* MW2 | 4044 | 91.8% (78/85) |
| >VFG001373 | cps4I, UDP-N-acetylglucosamine-2-epimerase | *Streptococcus pneumoniae* TIGR4 | 1098 | 91.8% (78/85) |
| >VFG001279 | sdrC, Ser-Asp rich fibrinogen-binding bone sialoprotein-binding protein | *Staphylococcus aureus* subsp. *aureus* MW2 | 2868 | 91.1% (82/90) |
| >VFG001281 | sdrE, Ser-Asp rich fibrinogen-binding bone sialoprotein-binding protein | *Staphylococcus aureus* subsp. *aureus* MW2 | 3426 | 91.1% (41/45) |
| >VFG002409 | essC, type VII secretion system protein EssC, FtsK/SpoIIIE family ATPase | *Staphylococcus aureus* subsp. *aureus* MW2 | 4440 | 81.7% (510/624) |

**Table S11 Antibiotic‐resistant genes in strains LVI A0006 and LVI A0039**

| Strain number | RGI Criteria | ARO Term | AMR Gene Family | Drug Class | Identity of Matching Region | Length of Reference Sequence |
| --- | --- | --- | --- | --- | --- | --- |
| LVI A0006 | Strict | vanY gene in vanF cluster | vanY, glycopeptide resistance gene cluster | glycopeptide antibiotic | 37.93 | 138.23 |
| LVI A0039 | Strict | vanY gene in vanF cluster | vanY, glycopeptide resistance gene cluster | glycopeptide antibiotic | 37.93 | 138.23 |

**Supplementary Figures**


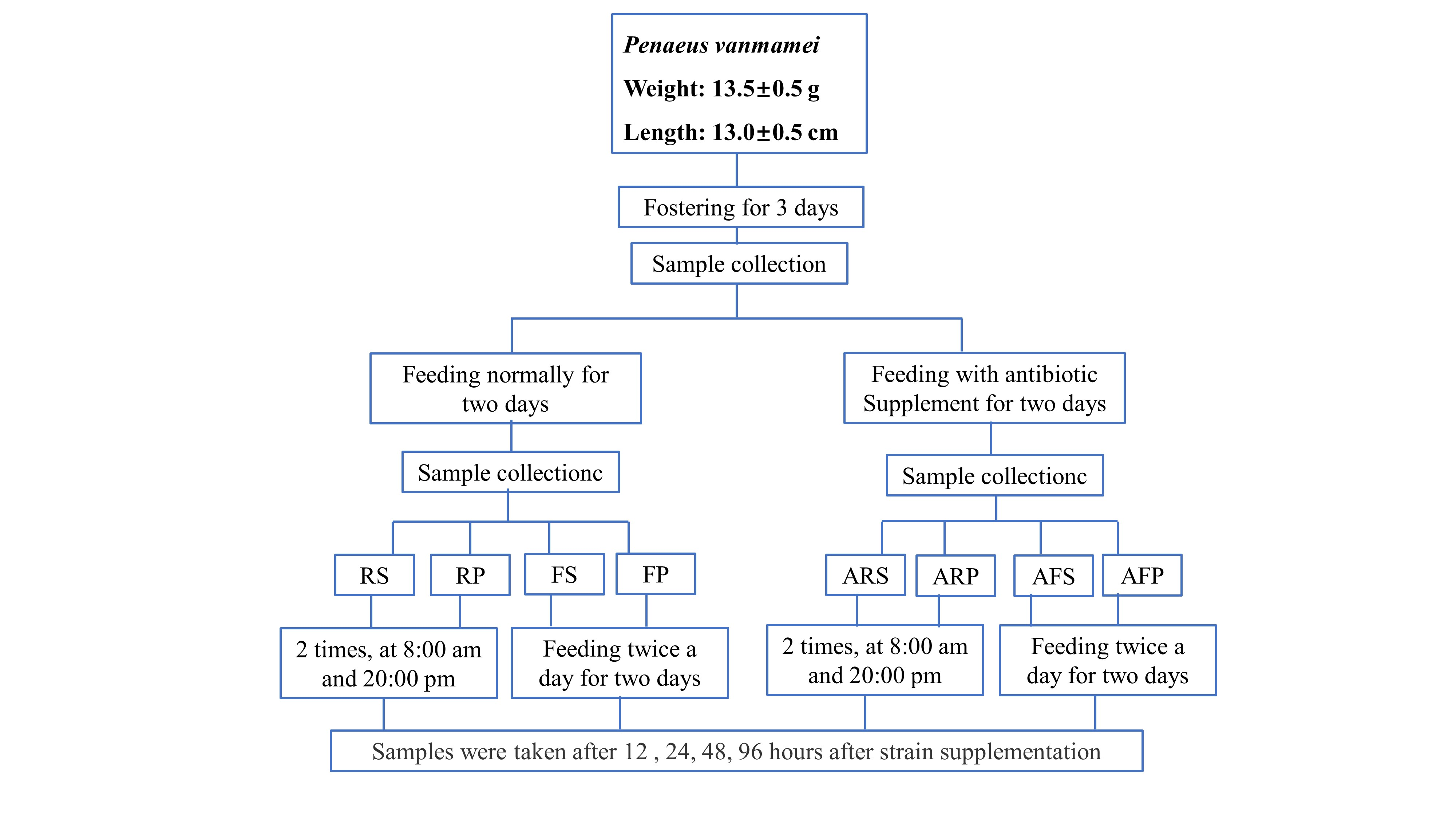


**Figure S1** The figure of experiment design for the function verification of strain LVI A0039


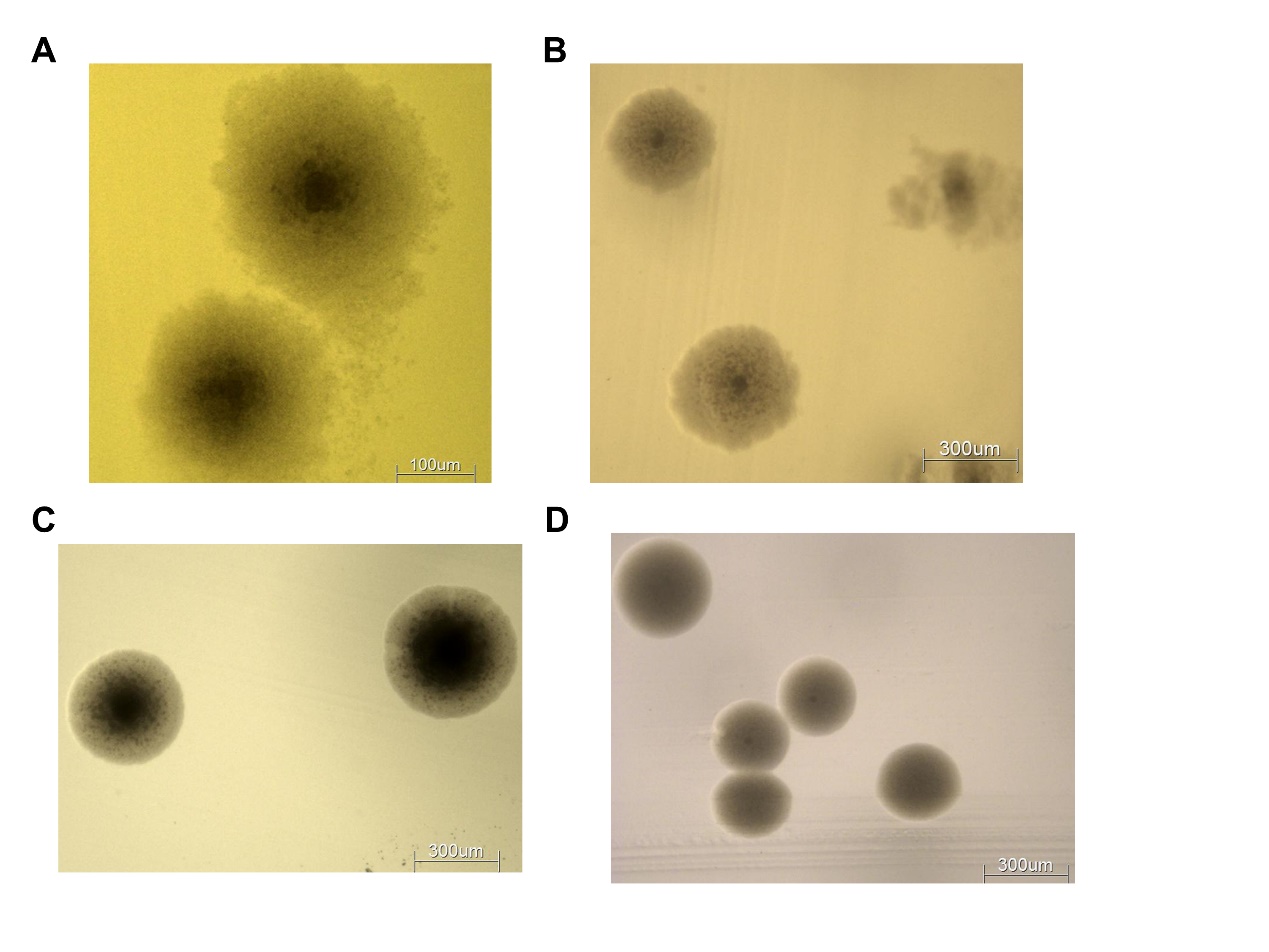


**Figure S2** Colony morphology of strains LVI A0006, LVI A0039, LVI A0075 and LVI A0078 cultured on solid PPLO medium for 3 days.


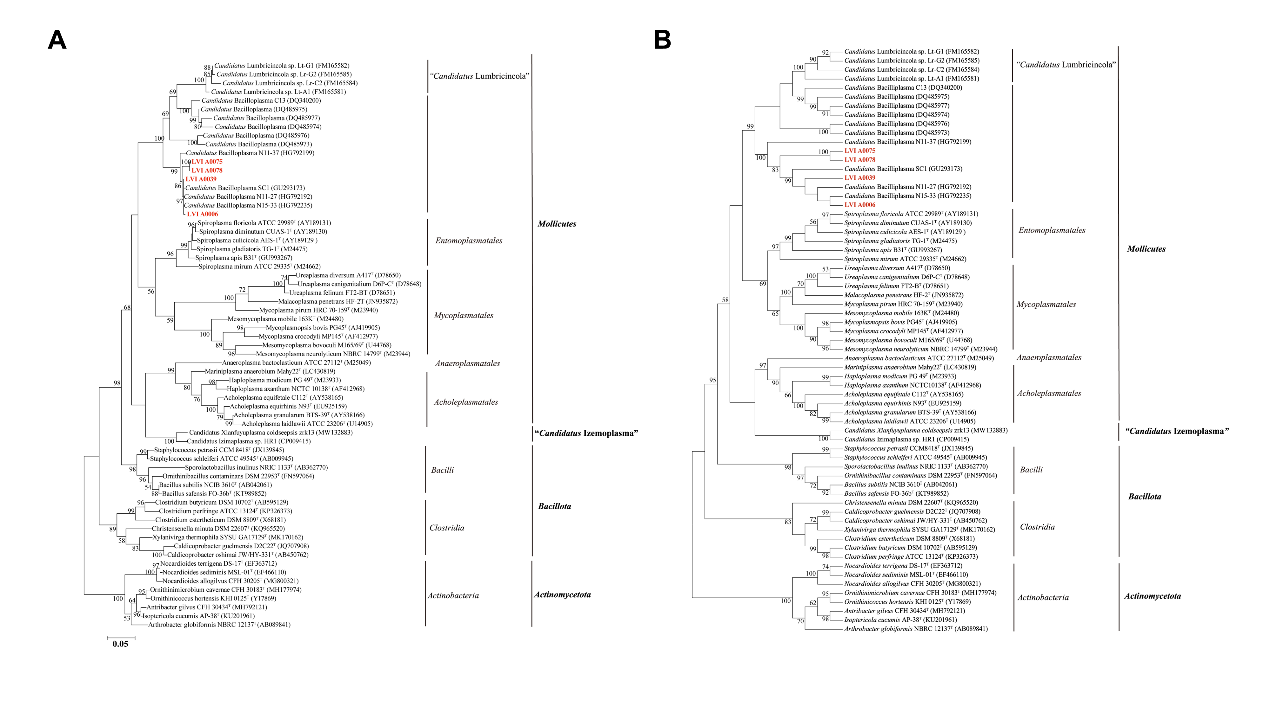


**Figure S3** Phylogenetic analysis of Strains LVI A0006, LVI A0039, LVI A0075 and LVI A0078. **(A).** Maximum-likelihood phylogenetic tree showing the relationship between the four strains and its closely related strains. **(B).** Maximum-parsimony phylogenetic tree showing the relationship between the four strains and its closely related strains. Bootstrap values (expressed as percentages of 1000 replications) of above 50 % are shown at the branch points.


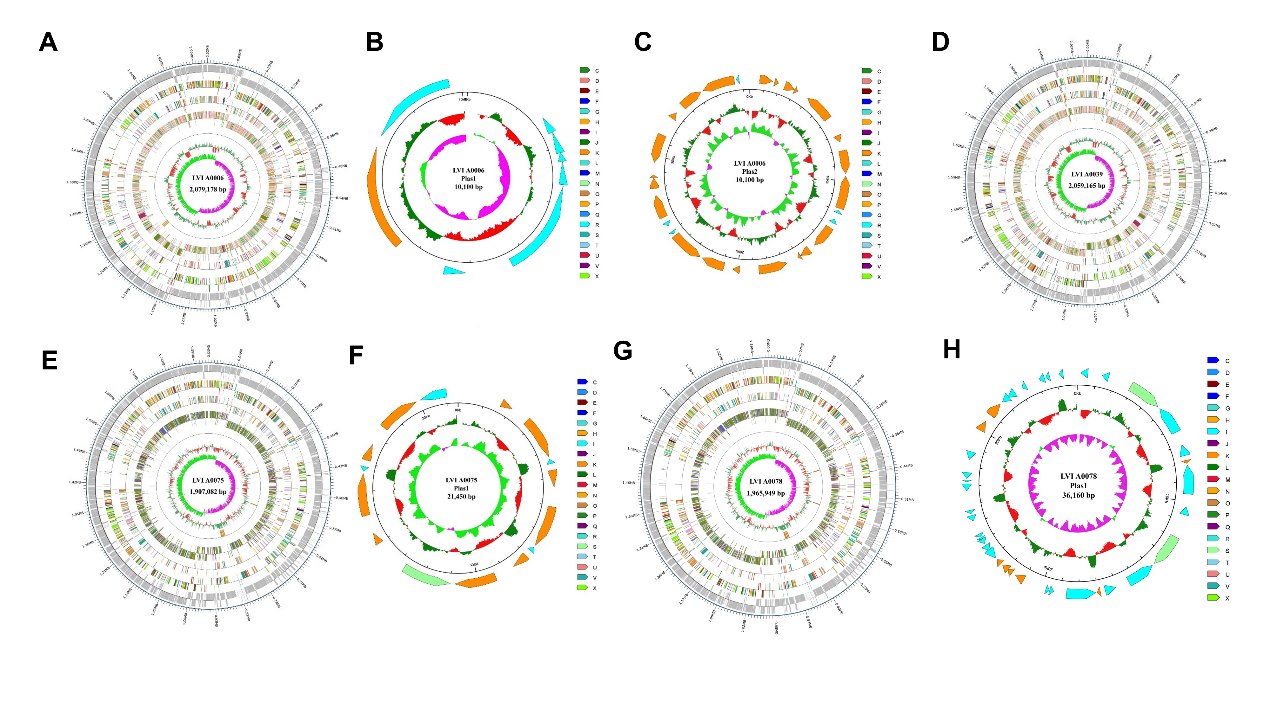


**Figure S4** Complete genome map of chromosome and plasmid of strains LVI A0006，LVI A0039，LVI A0075 and LVI A0078. **(A), (B) & (C).** complete genome map of chromosome and plasmid of strain LVI A0006; **d.** complete genome map of chromosome of strain LVI A0039, **(E) & (F).** complete genome map of chromosome and plasmid of strain LVI A0075; **(G) & (H).** complete genome map of chromosome and plasmid of strain LVI A0006.

Note: Rings indicate of chromosome genomic map, from outside to the center: sequence coordinates; coding gene; gene function annotation of COG, KEGG and GO database; ncRNA genes; GC content; GC skew. Rings indicate of plasmid genomic map, from outside to the center: gene function annotation of COG category, sequence coordinates, GC content, GC skew.


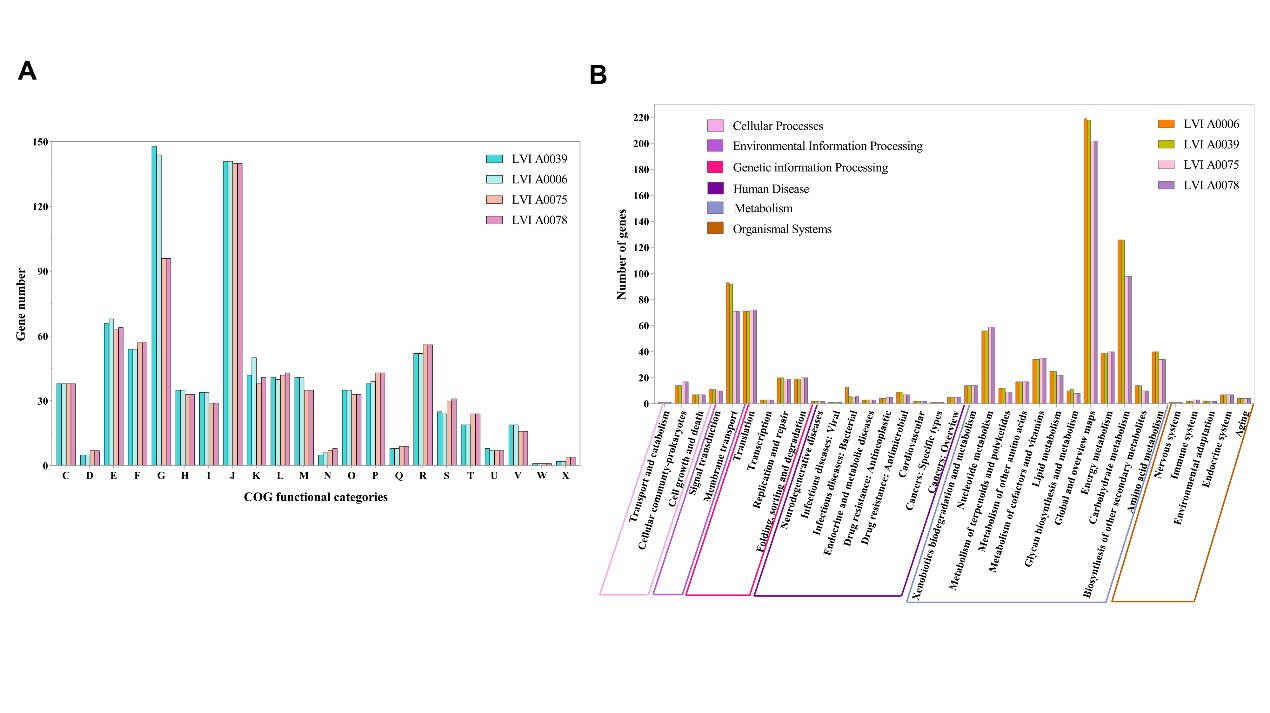


**Figure S5** Function annotations of COG (A) and KEGG (B) database in the genomes of four strains

**Description of novel members in Class *Mollicutes***

**Description of** ***Enteroplasmatales* ord. nov.**

*Enteroplasmatales* (N.L. neut. n. *Enteroplasma*, type genus of the order; L. fem. pl. n. suff. -ales, ending to denote an order; N.L. fem. pl. n. *Enteroplasmatales*, the *Enteroplasma* order).

This order belongs to the class Mollicutes, in the phylum *Mycoplasmatota*. Cells lack the cell wall and are bounded only by one plasma membrane, and some species can pass through 450 nm filters. Cells are Gram-stain-negative, non-motile, most cultivable species can grow well in serum-free media. Colonies on solid media are very small, usually much less than 1 mm in diameter. Under suitable conditions, almost all species form colonies that have a characteristic fried-egg appearance. No tricarboxylic acid cycle enzymes, quinones, or cytochromes have been found. The type genus is *Enteroplasma* gen. nov..

**Description of *Enteroplasmataceae* fam. nov.**

*Enteroplasmataceae* (N.L. neut. n. *Enteroplasma*, type genus of the family; L. fem. pl. n. suff. -aceae, ending to denote a family; N.L. fem. pl. n. *Enteroplasmataceae*, the *Enteroplasma* family). Cells lack a cell wall and are bounded only by a plasma membrane. Gram-stain-negative due to the absence of a cell wall, rod-shaped, usually nonmotile. The typical colony has a fried-egg, most cultivable species can grow well in serum-free media. Usually catalase-negative, lack of tricarboxylic acid cycle, quinones and cytochrome oxidase. The type genus is *Enteroplasma* gen. nov.

**Description of *Enteroplasma* gen. nov. and *Enteroplasma penaei* sp. nov., *Enteroplasma albus* sp. nov.**

For the genus name *Enteroplasma*, En.te.ro.plas.ma. Gr. neut. n. enteron, intestine; Gr. neut. n. plasma, something formed or molded, a form; N.L. neut. n. *Enteroplasma*, a bacterium of intestine. The genus *Enteroplasma* is a kind of obligate anaerobic microorganisms, whose cells are Gram-stain-negative, non-motile, non-spore-forming , rod-shaped and catalase-negative. Cells lack the cell wall and are bounded by one single plasma membrane, and some species can pass through 450 nm filters. Some have a specific tip structure that mediates attachment to host cells or other surfaces. This genus is a member of the Class *Mollicutes* (Edward and Freundt 1967) of the Phylum *Mycoplasmatota* (Murray 1984; Oren and Garrity 2021), The type species is *Enteroplasma penaei* LVI A0039.

*Enteroplasma penaei* (pe.na′e.i. N.L. n. *Penaeu*s a species of shrimp; N.L. gen. *penaei* of *Penaeus*, referring to *Penaeus vannamei*, from which the organism was isolated). Apart from the characters given for the genus, the species is characterized as follows. Have no catalase and oxidase activity. Cells are irregular rod-shaped and had no flagellum; obligate anaerobic; the temperature range for growth is 25°C-34°C with an optimum on 32°C; growing at pH values of 6.0-7.0; growth occurs at NaCl concentrations 0.0% up to 2.0%. Cells can pass through 450 nm filters. Cells can give positive results in tests for hydrolysis of gelatin but not in test for hydrolysis of urea. Utilizes α-D-Glucose, L-Fucose, L-Rhamnose, Esculin as the sole source of carbon in the API 20A tests. Weakly positive assimilation of α-D-lactose, D-Mannitol and D-Sucrose. No assimilation of D-Mannose, D-Raffinose, D-Sorbitol, D-Maltose, Salicin, Xylose, Arabinose, D-Cellose, Melezitose. Main fatty acids are C_14:0_FAME, C_18:1_CIS9FAME and C_16:0_FAME.

The type strain is LVI A0039, was isolated from the intestine of *Penaeus vanmamei* from shrimp culture ponds at Guangzhou city, Guangdong province, P. R. China. Its genome size and G+C content are 2,059,165 bp and 34.23 mol%, respectively.

*Enteroplasma albus* (alʹbus. L. masc. adj. *albus* white, referring to the dull-white color of colonies). Catalase and oxidase are negative. Cells are rod-shaped, and had no flagellum; obligate anaerobic; the temperature range for growth is 28°C-34°C with an optimum on 32°C; growing at pH values of 6.0-7.0; growth occurs at NaCl concentrations 0.0% up to 2.0%. Gives weakly positive results in tests for hydrolysis of gelatin but not in test for hydrolysis of urea. Utilizes α-D-Glucose, α-D-Lactose, D-Mannose, D-Raffinose, D-Mannitol, D-Sorbitol, L-Fucose, L-Rhamnose, D-Sucrose, D-Maltose, and Salicin as the sole source of carbon in the API 20A tests. Weakly positive assimilation of Arabinose and Glycerol. No assimilation of Xylose. Main fatty acids are C_16:0_ DMA, C_18:1_, C_11_or t9 or t6 FAME.

The type strain is LVI A0075^T^, was isolated from the intestine of *Penaeus vanmamei* from shrimp culture ponds at Guangzhou city, Guangdong province, P. R. China. Its genome size and G+C content is 1,907,082 bp and 34.14 mol%, respectively.
